# Fcγ Receptor I Alpha Chain (CD64) Expression in Macrophages Is Critical for the Onset of Meningitis by *Escherichia coli* K1

**DOI:** 10.1371/journal.ppat.1001203

**Published:** 2010-11-18

**Authors:** Rahul Mittal, Sunil K. Sukumaran, Suresh K. Selvaraj, David G. Wooster, M. Madan Babu, Alan D. Schreiber, J. Sjef Verbeek, Nemani V. Prasadarao

**Affiliations:** 1 Division of Infectious Diseases, The Saban Research Institute, Childrens Hospital Los Angeles, Los Angeles, California, United States of America; 2 Structural Studies Division, Medical Research Council, Laboratory of Molecular Biology, Cambridge, United Kingdom; 3 Hematology and Oncology Division, University of Pennsylvania School of Medicine, Philadelphia, Pennsylvania, United States of America; 4 Department of Human Genetics, University Medical Center, Leiden, Netherlands; 5 Keck School of Medicine, University of Southern California, Los Angeles, California, United States of America; Duke University Medical Center, United States of America

## Abstract

Neonatal meningitis due to *Escherichia coli* K1 is a serious illness with unchanged morbidity and mortality rates for the last few decades. The lack of a comprehensive understanding of the mechanisms involved in the development of meningitis contributes to this poor outcome. Here, we demonstrate that depletion of macrophages in newborn mice renders the animals resistant to *E. coli* K1 induced meningitis. The entry of *E. coli* K1 into macrophages requires the interaction of outer membrane protein A (OmpA) of *E. coli* K1 with the alpha chain of Fcγ receptor I (FcγRIa, CD64) for which IgG opsonization is not necessary. Overexpression of full-length but not C-terminal truncated FcγRIa in COS-1 cells permits *E. coli* K1 to enter the cells. Moreover, OmpA binding to FcγRIa prevents the recruitment of the γ-chain and induces a different pattern of tyrosine phosphorylation of macrophage proteins compared to IgG2a induced phosphorylation. Of note, FcγRIa^−/−^ mice are resistant to *E. coli* infection due to accelerated clearance of bacteria from circulation, which in turn was the result of increased expression of CR3 on macrophages. Reintroduction of human FcγRIa in mouse FcγRIa^−/−^ macrophages *in vitro* increased bacterial survival by suppressing the expression of CR3. Adoptive transfer of wild type macrophages into FcγRIa^−/−^ mice restored susceptibility to *E. coli* infection. Together, these results show that the interaction of FcγRI alpha chain with OmpA plays a key role in the development of neonatal meningitis by *E. coli* K1.

## Author Summary

*Escherichia coli* K1 is the most common cause of meningitis in premature infants; the mortality rate of this disease ranges from 5% to 30%. A better understanding of the pathogenesis of *E*. *coli* K1 meningitis is needed to develop new preventative strategies. We have shown that outer membrane protein A (OmpA) of *E. coli* K1, independent of antibody opsonization, is critical for bacterial entrance and survival within macrophages. Using a newborn mouse model, we found that depletion of macrophages renders the animals resistant to *E. coli* K1 induced meningitis. OmpA binds to α-chain of Fcγ-receptor I (FcγRIa) in macrophages, but does not induce expected gamma chain association and signaling. FcγRIa knockout mice are resistant to *E. coli* K1 infection because their macrophages express more CR3 and are thus able to kill bacteria with greater efficiency, preventing the development of high-grade bacteremia, a pre-requisite for the onset of meningitis. These novel observations demonstrate that inhibiting OmpA binding to FcγRIa is a promising therapeutic target for treatment or prevention of neonatal meningitis.

## Introduction

Professional phagocytes, including neutrophils and macrophages (MØ) express a specific set of phagocytic receptors that recognize, bind to and mediate internalization of microbial pathogens [1], [2], [3]. Although MØ receptor-mediated phagocytosis generally leads to the destruction of the pathogen, certain receptor-ligand interactions allow for a permissive environment in which the pathogen can thrive and even proliferate. MØ provide a barrier that pathogens must overcome to adhere to and penetrate into tissues. Nonetheless, diverse strategies are used by different bacterial pathogens to subvert phagocytes. *Escherichia coli* K1 causes meningitis in neonates, which remains a significant problem for the last few decades with case fatality rates ranging from 5 to 40% of infected neonates [4], [5], [6], [7]. Despite treatment with advanced antibiotics, up to 30% of survivors exhibit neurological sequelae such as hearing impairment, mental retardation, and hydrocephalus. Furthermore, due to the emergence of antibiotic resistant strains, mortality rates may significantly increase in future [8]. The crossing of the mucosal epithelium and the invasion of small subepithelial blood vessels by *E. coli* K1 represent critical early steps in the pathogenesis of meningitis. During initial colonization, *E. coli* K1 encounters several host defense mechanisms such as complement, neutrophils, and MØ on its path to the blood-brain barrier (BBB). However, very little is known about the mechanisms by which *E. coli* K1 finds a niche to avoid these host defenses. Our previous studies demonstrated that *E. coli* K1 evades complement attack by binding to the complement pathway regulator C4bp via outer membrane protein A (OmpA), which subsequently cleaves C3b and C4b complement proteins [9], [10]. In addition, lack of significant quantities of C9, a terminal complement component necessary for the formation of the membrane attack complex, in neonatal population gives an additional opportunity for *E. coli* K1 to survive in the blood [10]. However, our studies have shown that an inoculum of >10^3^ CFU/ml of *E. coli* K1 is required to resist serum bactericidal activity [11], indicating that the bacteria must take a refuge in certain cells to survive and multiply during the initial stages of infection, when fewer bacteria are present in the blood.

Despite the importance of MØ in innate and adaptive immunity, the interaction of *E. coli* K1 with these cells is poorly defined. MØ phagocytose a broad range of pathogens by recognizing pathogen-associated molecular pattern (LPS and peptidoglycans) via pattern recognition receptors (PRR), which include TLRs, the mannose receptor and the scavenger receptor [12], [13]. Opsonin-dependent phagocytosis involves complement receptors and antibody-dependent phagocytosis requires Fcγ receptors. Studies from our lab have shown that *E. coli* K1 enters and multiplies in both human and murine MØ, either in the presence or absence of opsonization. OmpA expression is critical for these processes [14]. Therefore, it is important to determine whether *E. coli* OmpA interacts with any cell surface proteins of MØ for entry. Numerous studies have shown that the expression of FcγRI is increased during septicemia and meningitis caused by a variety of pathogens [15], [16], [17], although its importance in any of these infections has not been explored. Fcγ receptors (FcγR) recognize the Fc region of IgG and play a pivotal role in linking the cellular and humoral arms of the immune response. FcγR comprises a multigene family divided into 3 classes (FcγRI, II and III), which are defined by their affinity for IgG [18], [19], [20], [21], [22], [23]. FcγRI is a transmembrane receptor, which binds IgG with high affinity and induces the association of the γ-chain for signal transduction and triggering of effector responses such as MØ phagocytosis [23]. The ligation of FcγRI with IgG also mediates antibody-dependant cellular cytotoxicity induced transcription of cytokine genes and release of inflammatory mediators [24]. The cytoplasmic domain of the γ-chain contains an immunoreceptor tyrosine activation motif (ITAM), which is necessary for the signaling cascade of FcγRs. The cytoplasmic domain of FcγRI has been shown to modulate receptor function, although it does not contain any recognized signaling motif [25], [26]. In this study, we examined the role of MØ and FcγRI α-chain (FcγRIa) in the pathogenesis of neonatal *E. coli* K1 meningitis by depleting MØ from newborn mice and utilizing a FcγRIa^−/−^ knockout mouse model. Our studies provide evidence of a role for a novel interaction between FcγRIa and OmpA in the onset of meningitis due to *E. coli* K1.

## Results

### MØ-depleted newborn mice are resistant to *E. coli* K1 induced meningitis

**Figure 1:**
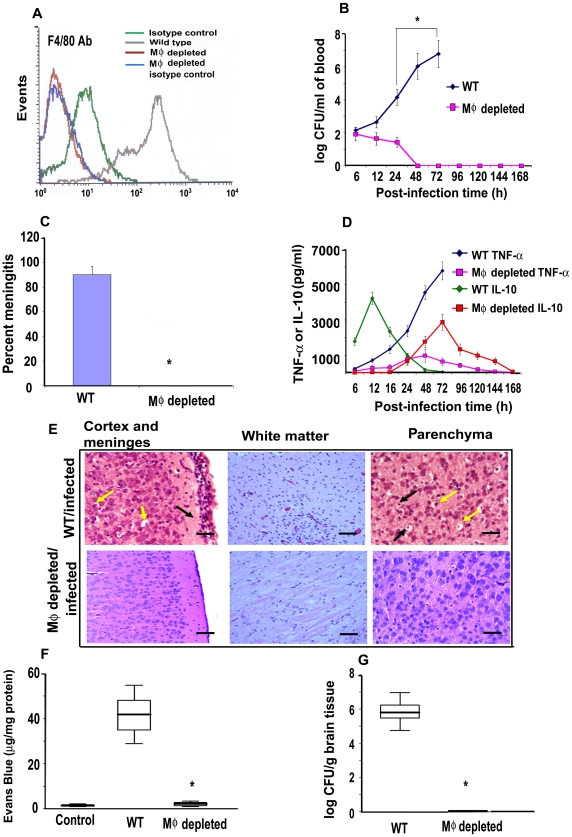
Depletion of MØ in newborn mice prevented the occurrence of meningitis by *E. coli* K1. Newborn mice were administered α-carrageenan once a day for three days after birth. Spleens and livers were harvested, homogenized and the cells in the homogenates were subjected to flow cytometry after staining with F4/80 antibodies. Cells from untreated animals and those stained with isotype-matched antibodies were used as controls (A). The MØ-depleted animals were infected with 10^3^ CFU of *E. coli* K1 by intranasal instillation and blood was collected at different post-infection times. Various dilutions of the blood were plated on agar containing rifampicin (B). Cerebrospinal fluid was collected from the same animals aseptically by cisternal puncture and inoculated into LB broth containing antibiotics (C). Blood collected from these animals was also used to measure the presence of TNF-α or IL-10 by ELISA (D). At 72 h post-infection, animals were sacrificed due to a moribund situation for ethical reasons, and the brains were harvested, fixed, paraffin sections prepared and stained with Hematoxylin and Eosin (E). Neutrophil infiltration (black arrow) was observed in the cortex and meninges in brains of WT mice along with apoptosis of neurons indicated by perinuclear halo (yellow arrows). White matter showed increased cellularity due to inflammatory exudates. Pkynotic nuclei (yellow arrows) and inflammatory cells (black arrow) were observed in the hippocampus, suggesting apoptosis of neurons. In contrast, no such pathological changes were seen in the brains of MØ-depleted mice. In some experiments, the animals were injected intraperitoneally with Evans blue at 68 h post-infection. The animals were sacrificed at 72 h the brains were harvested, then homogenized and the concentration of Evans blue determined (F). Brain homogenates from uninfected animals were used as controls. Half of the brain from each animal was homogenized and the presence of *E. coli* K1 determined by plating the homogenates on antibiotic containing agar (G). The data represent mean values ± SE of three separate experiments with a total of 15 animals per group. Histopathology is from one animal that is representative of similar results from the rest of the experimental group. Blood brain barrier leakage and the bacterial burden in MØ-depleted animals were statistically different when compared with the untreated and infected animals, *p<0.001 by Student's *t* test. Scale bars, 20 µM.

Our previous studies have shown that OmpA+ *E. coli* enters and survives in human and mouse MØ [14]. To determine the role of MØ in the pathogenesis of *E. coli* K1 induced meningitis, MØ were depleted in newborn mice by the administration of carrageenan [27], [28]. MØ readily ingest carrageenan in contrast to lymphocytes, which are not actively phagocytic and lack a well-developed lysosomal complex. Due to its unique secondary and tertiary structure, carrageenan is resistant to biochemical degradation by lysosomal glycosidases. Carrageenan containing phagolysosomes eventually rupture due to osmotic swelling. The consequent release of hydrolytic enzymes into the cytosol causes irreversible damage and eventual lysis of MØ [29]. Following three days of carrageenan administration starting at day 1 after birth, the animals showed >95% depletion of MØ from livers and spleens, as shown by flow cytometry after staining with F4/80 antibody (5.33%±0.4% before and 0.17%±0.1% after carrageenan treatment) (Figure 1A). However, treatment with carrageenan did not affect B cells (39.81%±0.7% before and 40.19%±0.9% after carrageenan treatment), CD4+ T cells (17.56%±0.5% before and 18.02%±0.6% after carrageenan treatment), CD8+ T cells (2.11%±0.4% before and 2.53%±0.3% after carrageenan treatment), DCs (5.67%±1.2% before and 6.09%±0.9% after carrageenan treatment), or PMNs (3.98%±1.2% before and 4.13%±1.4% after carrageenan treatment) in spleens of MØ-depleted mice compared with untreated mice (Figure S1). The MØ-depleted mice were then infected with 10^3^ CFU of *E. coli* K1 by intranasal instillation and examined for progression of the disease as previously described [28]. Control animals (n = 15 for each group) developed bacteremia at 6 h post-infection, which was increased to 5.5 log_10_ CFU per ml of blood by 48 h (Figure 1B). In contrast, the MØ-depleted mice, despite having a similar number of bacteria in the blood at 6 h, cleared these bacteria from the circulation by 48 h post-infection. In agreement with the bacteremia levels, >90% of control mice developed meningitis at 72 h after infection, whereas none of the MØ-depleted animals showed signs of meningitis and all survived beyond 7 days (Figure 1C). Determination of serum cytokine levels at various times post-infection revealed that control animals produced an initial burst of IL-10, which peaked at 12 h, and then declined to basal levels by 48 h (Figure 1D). In contrast, the pro-inflammatory cytokines, TNF-α, IFN-γ, IL-1β, IL-6 and IL-12p70 only became detectable in the blood at 12 h post-infection and peaked by 72 h (Figure 1D and Figure S2). Of note, although the MØ-depleted mice had early production of pro-inflammatory cytokines, their levels were significantly lower than those in the control mice. In these mice, IL-10 levels progressively rose during the initial stages of infection and peaked at 72 h at which time the bacteria were completely cleared from the circulation (Figure 1D).

Histopathological examination of control mice infected with *E. coli* K1 revealed marked infiltration of PMNs in the leptomeningeal and ventricular spaces (Figure 1E). The hippocampus was also inflammed and there was apoptosis of neurons, as indicated by pkynotic nuclei in Ammon's horn. Acute hemorrhage and inflammation was observed, most prominently in the white matter of the brain. The cortex and molecular layer had increased cellularity due to inflammatory exudates. The MØ-depleted mice, however, did not reveal such pathological changes. Blood brain barrier (BBB) leakage is the hallmark of neonatal meningitis. Therefore we used the Evans blue extravasation method to quantify BBB leakage in both the control and MØ**-**depleted mice [28]. The dye was injected intraperitoneally at 68 h post-infection and after four hours, the brains were removed and Evans blue concentration determined. A marked increase in the permeability of the BBB was observed in infected WT animals, which was significantly reduced in MØ-depleted mice, (p<0.001 by student's *t* test) (Figure 1F). Furthermore, the number of *E. coli* K1 entering the brain was approximately 6.0 log_10_ CFU in control animals, whereas the brains of the MØ-depleted animals contained very few bacteria (Figure 1G). These results demonstrate that MØ may be important for *E. coli* K1 to reach a required level of bacteremia, which is critical for the establishment of neonatal meningitis.

### OmpA interaction with FcγRIa is requisite for *E. coli* K1 binding to, and entry into, MØ

**Figure 2:**
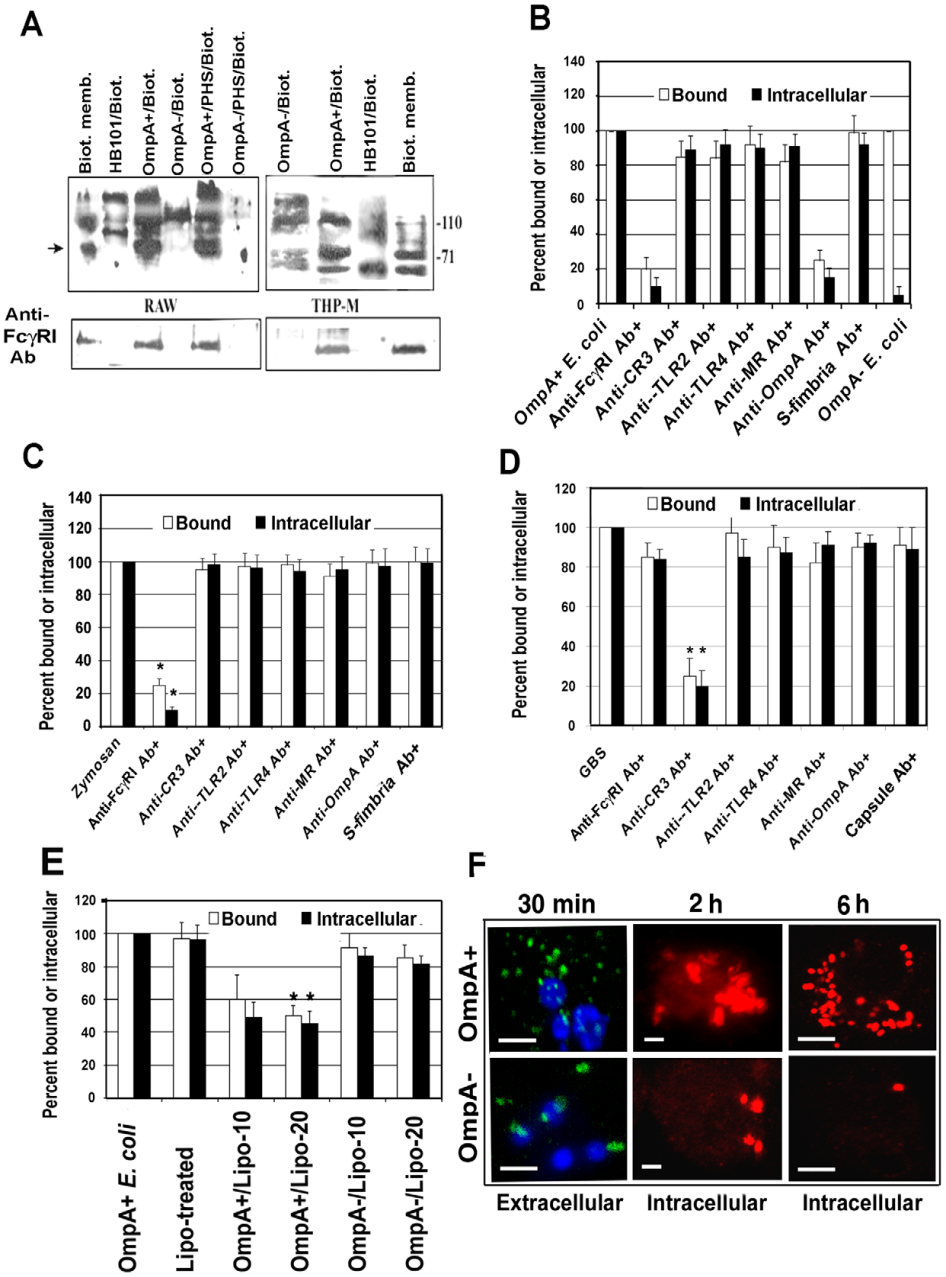
OmpA interaction with FcγRIa is necessary for binding to, and entry of, *E. coli* K1 in RAW 264.7 cells. (A) The surface proteins of RAW 264.7 cells and THP-1 differentiated into macrophages (THP-M) were labeled with NHS-LC-Biotin and the membrane proteins prepared. OmpA+ or OmpA− *E. coli*, with or without treatment with 40% pooled human serum for 10 min, or HB101 were incubated with 2 µg of biotinylated proteins for 1 h, washed, the bound proteins released, and subjected to SDS-PAGE. The proteins were then transferred to a nitrocellulose and immunoblotted with streptavidin peroxidase. The blots were stripped and reprobed with anti-FcγRI antibody. (B) RAW 264.7 cells were incubated with various antibodies prior to the addition of *E. coli* K1. Similarly, OmpA+ *E. coli* were incubated with anti-OmpA antibodies for 1 h on ice prior to adding to MØ. Isotype-matched antibodies or anti-S-fimbria antibodies were used as controls. Bound and intracellular bacteria were determined by the gentamicin protection assay as described in Materials and Methods. Bound or intracellular bacteria of untreated cells were taken as 100%. (C and D) Similar studies were also performed with Zymosan coated with IgG2a and Group B *streptococcus*. Isotype-matched antibodies or anti-capsular antibodies were used as controls. (E) Liposomes containing OmpA or outer membrane proteins of OmpA− *E. coli* were incubated with RAW 264.7 cells for 30 min prior to adding the bacteria. Relative total cell bound and intracellular bacteria (by gentamicin protection assay) were determined. (F) RAW 264.7 cells were infected with OmpA+ or OmpA− *E. coli* for various time points, washed, fixed and differentially stained for extracellular and intracellular bacteria using anti-S-fimbria antibodies. The extracellular bacteria were stained with FITC-coupled secondary antibodies (Green) while the intracellular bacteria were stained with Cy3-coupled secondary antibodies (Red). Scale bars, 10 µM. Data shown are mean values ± SD of three separate experiments performed in triplicate. The reduction in bound or invaded bacteria was statistically significant compared with control untreated and infected cells, *p<0.001 by Student's *t* test.

Our previous studies have shown that OmpA+ *E. coli* binds and enters MØ *in vitro* irrespective of opsonization status of the bacteria [14]. OmpA− *E. coli*, although entered in lower numbers but failed to survive inside MØ. This indicates that OmpA mediated entry into MØ enables OmpA+ *E. coli* K1 to resist the normal antimicrobial mechanisms of MØ. Therefore, to understand the nature of the macrophage surface structures that interact with *E. coli* K1, biotin-labeled cell surface proteins of THP-1 cells differentiated into MØ (THP-M) and RAW 264.7 cells were incubated with OmpA+ *E. coli*, OmpA− *E. coli* or a laboratory *E. coli* HB101. Bound proteins were then released and analyzed by western blotting with streptavidin peroxidase. A small number of proteins bound to all the bacteria from both the cells. However, OmpA+ *E. coli* prominently bound to the 110 and 70 kDa proteins from both THP-M and RAW 264.7 cells, whereas OmpA− *E coli* bound only to the 110 kDa protein (Figure 2A). Although some proteins bound to HB101 were of similar molecular mass to those bound to OmpA+/OmpA− *E. coli*, other proteins showed different binding patterns. Based on their molecular masses, we speculated that the proteins binding to *E. coli* K1 could be Toll like receptor-4 (110 kDa) and FcγRIa (CD64, 72 kDa). Since OmpA+ *E. coli* specifically bound to the 70 kDa protein in contrast to OmpA− *E. coli*, the blots were reprobed with an anti-FcγRI antibody, which reacted with the 70 kDa protein, suggesting that OmpA+ *E. coli* binds to FcγRIa. Of note, treating the bacteria with 40% pooled human serum did not alter the binding, indicating that opsonization with complement and/or with non-specific antibody did not alter bacterial interaction with macrophage surface proteins.

Next, we used blocking antibodies to determine the contribution of OmpA-FcγRIa interaction in *E. coli* entry into MØ. OmpA+ *E. coli* was incubated with Fab fragments of anti-OmpA antibody (polyclonal) prior to addition to MØ. In other experiments, the RAW 264.7 cells were pre-treated with antibodies to FcγRI, CR3, TLR2, TLR4 or the mannose receptor prior to addition of OmpA+ *E. coli*. Isotype matched antibodies or anti-S-fimbria antibodies were used as controls. Both anti-OmpA and anti-FcγRI antibodies reduced the number of bound and intracellular *E. coli* K1 by ∼80%, whereas other antibodies showed no significant inhibition (Figure 2B). To verify that the anti-FcγRI antibody actually inhibited FcγRI–mediated phagocytosis, the effect of this antibody on the entry of zymosan coated with fluorescent-labeled IgG2a that occurs via FcγRI was also determined. The internalized zymosan particles were counted per 100 cells after quenching the external fluorescence by Trypan Blue [30]. As predicted, anti-FcγRI antibodies significantly inhibited the entry of opsonized zymosan (Figure 2C).

MØ pretreated with the anti-FcγRI antibody were also infected with Group B *streptococcus* (GBS) pre-treated with C8-deficient serum (for deposition of C3 and to avoid bacterial killing by serum), which is known to enter MØ through the CR3 receptor [31], [32], [33]. The internalization of GBS, however, was not affected by pretreatment with anti-FcγRI antibody, suggesting that it did not interfere with CR3 receptor function in MØ (Figure 2D). However, as expected, anti-CR3 antibodies significantly blocked the binding and entry of GBS into RAW 264.7 cells. To further confirm the role of OmpA interaction with MØ in *E. coli* entry into MØ, OmpA was purified from OmpA+ *E. coli* and reconstituted into liposomes as previously described [34], which were used to pre-treat RAW 264.7 cells prior to adding the bacteria (Figure 2E). The liposomes containing OmpA blocked both binding and intracellular survival of *E. coli* K1 by approximately 50%, whereas liposomes containing outer membrane proteins from OmpA− *E. coli* did not show such inhibition. Increasing concentrations of OmpA liposomes showed no further increase in the inhibition, indicating that the structure of OmpA in liposomes may not be optimal to that of OmpA on *E. coli* K1 to bind to FcγRIa.

The fate of OmpA+ *E. coli* after phagocytosis by RAW 264.7 cells was examined by immunocytochemistry after differential staining. Extracellular bacteria were stained with FITC labeled secondary antibody (green) and the intracellular bacteria were stained with a TRITC labeled secondary antibody (red) after incubation with primary anti-S-fimbria antibody. As shown in Figure 2F, a number of OmpA+ *E. coli* bound to RAW 264.7 cells, whereas very few OmpA− *E. coli* bound at 30 min post-infection. Analysis of intracellular bacteria over time revealed that OmpA+ *E. coli* multiplied, whereas OmpA− *E. coli* were degraded inside the cells. Collectively, these studies suggest that the OmpA of *E. coli* K1 interacts with regions of FcγRIa similar to those involved in the binding of Fc and that this interaction enables the organism to enter MØ. In addition, the data suggest that other receptors that recognize pathogen-associated molecules may not play a significant role in MØ binding and entry of *E. coli* K1. However, entry through other receptors in the absence of OmpA-FcγRIa interaction renders the bacteria susceptible to macrophage killing.

### FcγRIa gene silencing by RNA interference abolishes *E. coli* K1 binding to and entry into MØ

**Figure 3:**
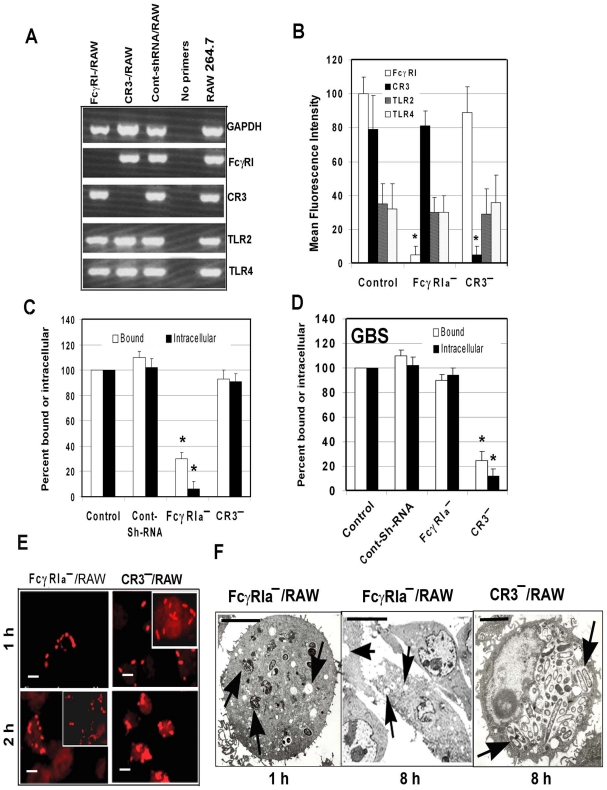
Suppression of FcγRIa expression using shRNA prevents *E. coli* K1 entry into RAW 264.7 cells. (A) RAW 264.7 cells were transfected with plasmids containing shRNA to FcγRIa or CR3, total RNA was isolated and subjected to RT-PCR using specific primers. GAPDH primers were used as internal controls. (B) FcγRIa^−^/− and CR3^−^/RAW cells were further subjected to flow cytometry using antibodies to FcγRI, CR3, TLR2 and TLR4. Mean fluorescence intensities were plotted after subtracting the values of isotype-matched controls. (C and D) Total cell bound and intracellular bacteria (measured by gentamicin protection assay) were determined after infecting FcγRIa^−^/− and CR3^−^/RAW cells with *E. coli* K1 or Group B *streptococcus. E. coli* K1 or GBS that were bound or intracellular in control cells were taken as 100%. (E) Immunocytochemistry of *E. coli* K1 entered into FcγRIa^−^/− and CR3^−^/RAW cells after differential staining as described in Materials and Methods. Scale bars, 10 µM. (F) FcγRI^−/−^ and CR3^−^/RAW cells were infected with *E. coli* K1 for varying periods, fixed and subjected to transmission electron microscopy as described in Materials and Methods. Photomicrographs at 1 h and 8 h post-infection are shown and arrows indicate vacuoles containing bacteria or empty vacuoles. Invasion experiments were performed in triplicate and were independently done three times. Data represent mean ± SD and the decrease in bound or intracellular bacteria was statistically significant when compared with control shRNA/RAW 264.7cells, *p<0.001 by Student's *t* test. Scale Bars 1.0 µM.

To confirm the role of FcγRIa in OmpA+ *E. coli* entry of MØ, short hairpin RNA (shRNA) sequences for murine FcγRIa and CR3 in pGeneClip Neomycin vectors were used to transfect RAW 264.7 cells. Suppression of FcγRIa and CR3 gene transcription and expression was verified by RT-PCR and flow cytometry, respectively. The respective shRNA suppressed the transcription of FcγRIa and CR3 considerably, but had no effect on GAPDH, TLR2 or TLR4 mRNA transcript levels (Figure 3A). On par with changes in transcription levels, the surface expression of FcγRIa and CR3 was significantly reduced, while TLR2 and TLR4 expression was unaltered (Figure 3B). There was >90% reduction in the OmpA+ *E. coli* phagocytosed by FcγRIa-shRNA/RAW cells compared to control or CR3-shRNA/RAW cells (p<0.001 by two-tailed *t* test) (Figure 3C). This reduction was due to inefficient binding of *E. coli* K1 to these cells, as less than 30% of bacteria were bound by the FcγRIa-shRNA/RAW cells compared to non-transfected or control-shRNA transfected cells. In contrast, both binding and intracellular survival of GBS were not affected in FcγRIa-shRNA/RAW cells, whereas CR3-shRNA transfection caused significant reduction in both of these processes (Figure 3D). Immunocytochemistry of *E. coli* K1 infected FcγRIa-shRNA/RAW cells revealed that very few cells ingested bacteria and were killed within 2 h post-infection (Figure 3E, fragmented bacteria). However, *E. coli* K1 entered and replicated in CR3-shRNA/RAW cells similar to control RAW cells. Comparable results were also obtained with THP-M cells transfected with shRNA specific to human FcγRI (data not shown). To further confirm that lack of FcγRIa expression rendered bacteria susceptible to macrophage killing, FcγRIa-shRNA/RAW cells infected with *E. coli* K1 were examined by transmission electron microscopy. Although few numbers of FcγRIa-shRNA/RAW cells engulfed *E. coli* K1, several of them were either degraded or in the process of degradation by 1 h post-infection and were completely killed by 8 h post-infection (Figure 3F). In contrast, CR3-shRNA/RAW cells showed intact bacteria in endosomes undergoing significant multiplication by 8 h post-infection. Taken together these results demonstrate that OmpA-FcγRIa interaction is critical for *E. coli* K1 to bind to, enter and survive in MØ.

### COS-1 cells expressing FcγRIa are susceptible to *E. coli* K1 infection

**Figure 4:**
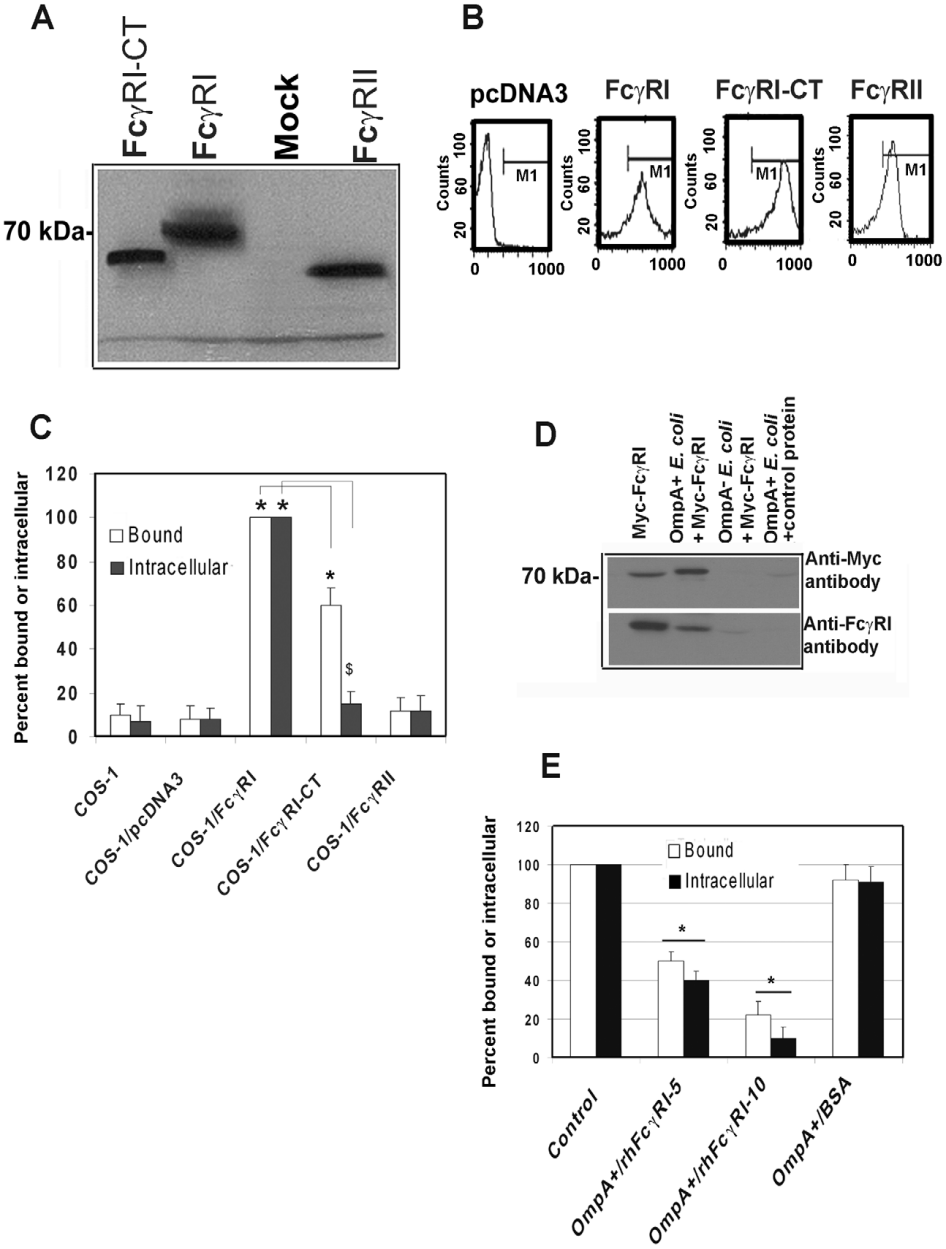
FcγRIa expression is sufficient to facilitate *E. coli* K1 invasion of COS-1 cells. COS-1 cells were transfected with plasmids containing FcγRIa, FcγRI-CT or FcγRII and the expression of the recombinant proteins was determined by Western blotting (A) from total cell lysates or by flow cytometry (B) using anti-Myc antibody. COS-1 cells transfected with pcDNA3 were used as a control (Mock). *E. coli* K1 binding to, and invasion of, transfected COS-1 cells were performed as described in Materials and Methods (C). Purified Myc-FcγRIa or BSA (control) was incubated with OmpA+ or OmpA− *E. coli* for 1 h on ice. Bacteria were washed, the bound proteins were released and were subjected to SDS-PAGE. The proteins were transferred to a nitrocellulose and immunoblotted with anti-Myc antibodies. In separate experiments, the bound proteins were blotted with anti-FcγRIa antibodies (D). Purified Myc-FcγRIa (5 and 10 µg) or BSA (10 µg) were incubated with OmpA+ *E. coli* separately, washed and then added to COS-1 cells. Total cell bound and intracellular bacteria were determined (E). Binding and invasion assays were performed at least three times in triplicate and the data represent mean ± SD. The increase or decrease in binding or invasion of *E. coli* was statistically significant compared to controls, *p or ^$^p<0.001 by Student's *t* test.

The activation of FcγRI in phagocytic cells by the binding of the Fc region of IgG requires the association of FcγRIa with the IgG γ-chain [19]. To examine whether γ-chain association with FcγRI is also necessary for *E. coli* K1 invasion, COS-1 cells were transfected with pcDNA3 plasmids containing Myc-tagged human FcγRIa (Myc-hFcγRIa), C-terminal truncated Myc-hFcγRIa which lacks the cytoplasmic tail (CT) or Myc-hFcγRII. Expression of these proteins was verified by Western blotting using the anti-Myc antibodies (Figure 4A) and flow cytometry (Figure 4B). OmpA+ *E. coli* binding to, and invasion of, hFcγRIa^+^/COS-1 cells was significantly greater compared to that of mock-transfected cells (Figure 4C). OmpA− *E. coli* showed very negligible binding to, and invasion into, both FcγRIa transfected and mock-transfected COS-1 cells (data not shown). The invasion of *E. coli* K1 into Myc-hFcγRIa-CT/COS-1 cells was significantly reduced, although the binding of bacteria to these cells was decreased by only 30% compared to Myc-hFcγRIa^+^/COS-1 cells. In contrast, overexpression of FcγRII did not increase *E. coli* binding to, or invasion of, COS-1 cells. These data suggest that FcγRIa acts as receptor for OmpA mediated entry of *E. coli* K1 into COS-1 cells and that the C-terminal portion is required for this invasion.

Next, to examine whether FcγRIa interacts with OmpA, recombinant hFcγRIa (rhFcγRIa) was purified by Myc-affinity column chromatography from COS-1 cells and incubated with OmpA+ or OmpA− *E. coli*. The bound proteins were released and subjected to Western blotting with antibodies to Myc or FcγRI. The purified rhFcγRIa bound to OmpA+ *E. coli* but not to OmpA− *E. coli*, whereas BSA, used as a control, did not interact with the bacteria (Figure 4D). rhFcγRIa used to pre-treat bacteria prior to adding them to COS-1 monolayers in the invasion assays resulted in much more significant inhibition of *E. coli* K1 binding to, and entry into the cells in a dose dependent manner when compared to the BSA control (Figure 4E). These results suggest that the alpha chain of FcγRI is sufficient for *E. coli* K1 to bind to, and invade, COS-1 cells.

### OmpA of *E. coli* K1 binds to FcγRIa and induces a distinct signaling pattern

**Figure 5:**
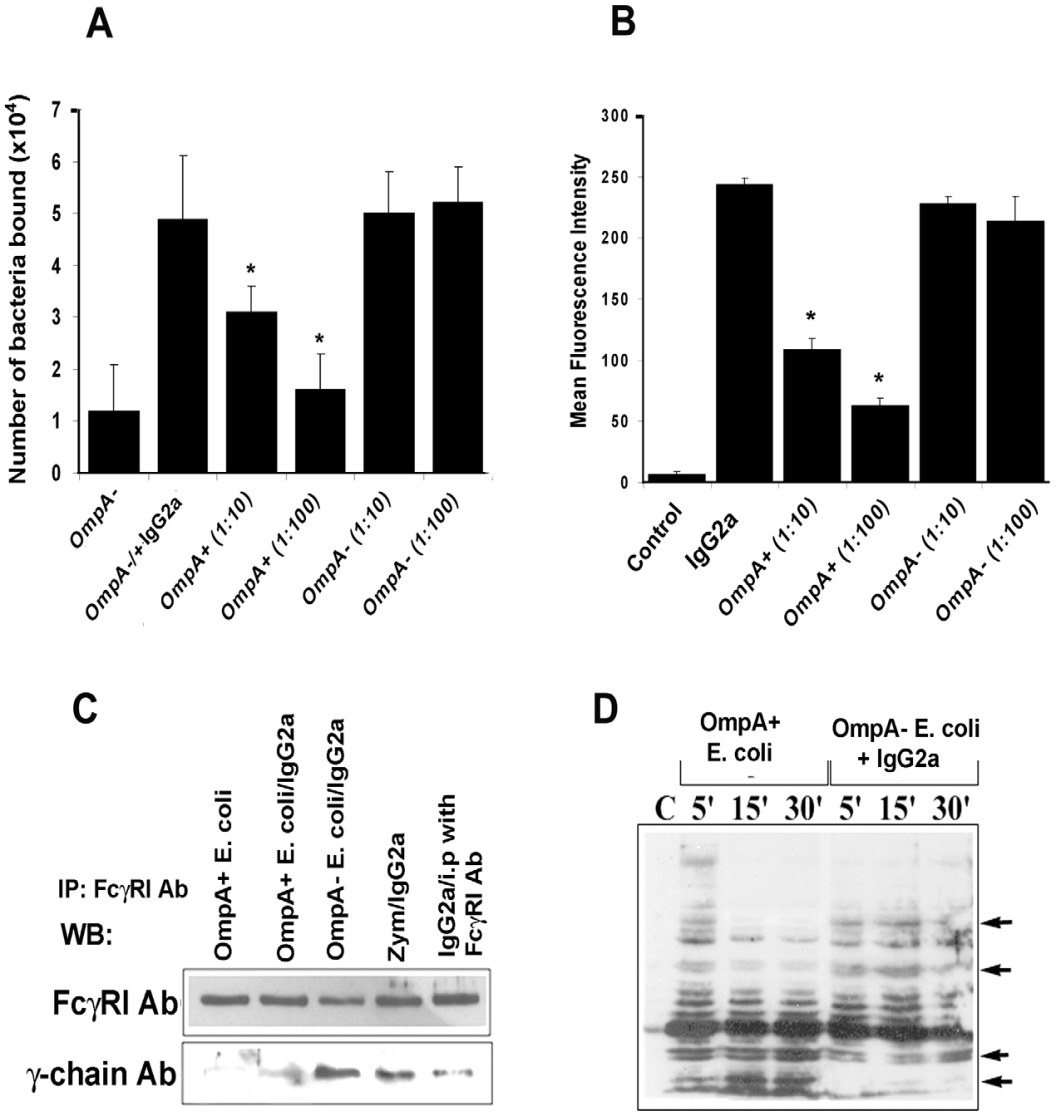
*E. coli* K1 binds to FcγRIa via OmpA and induces a distinct signaling. (A) OmpA− *E. coli* were coated with IgG2a for 1 h on ice, washed and then added to COS-1 cells pre-treated with cytochalasin D. After one hour of incubation, the cells were washed and OmpA+ *E. coli* were added at an MOI of 10 and 100. The cells were incubated for 10 min, washed, and the bound OmpA− *E. coli* enumerated as described in Materials and Methods. (B) Peritoneal MØ pre-treated with cytochalasin D were incubated with FITC-IgG2a for 30 min, washed, and further incubated with OmpA+ or OmpA− *E. coli* at an MOI of 10 or 100 for 10 min. The cells were washed and subjected to flow cytometry to determine the bound levels of IgG2a. Cells without the addition of IgG2a were used as a control. (C) Immunoprecipitation of total cell lysates obtained from RAW 264.7 cells infected with OmpA+ *E. coli*, OmpA− *E. coli* or Zymosan with anti- FcγRI antibody was followed by Western blotting with antibodies to γ-chain or FcγRI. (D) Total cell lysates of RAW 264.7 cells infected with OmpA+ or OmpA− *E. coli* were subjected to Western blotting with anti-phospho-tyrosine antibodies. Competitive inhibition studies were performed at least four times in triplicate and the data represent mean ± SD. The decrease in the number of bacteria attached to COS-1 cells or MFI was statistically significant compared to IgG2a coated OmpA− *E. coli*, *p<0.001 by Student's *t* test.

One important question to address in these studies is how OmpA of *E. coli* K1 binds to FcγRIa at the same region as the Fc-portion of IgG in the context of whole blood. Generally, specific or even non-specific IgG in circulation binds invading bacteria and thereby presents the pathogen to FcγR receptors on MØ. Therefore, it is possible that OmpA+ *E. coli* may be displacing IgG for binding to FcγRI. We tested this hypothesis by performing two different competitive binding experiments. First, OmpA− *E. coli* were coated with anti-S-fimbria antibody and added to FcγRIa^+^/COS-1 cells treated with cytochalasin D to prevent internalization. The cells were washed and then various quantities of OmpA+ *E. coli* were added and incubated for 10 min. After washing the monolayers, the number of OmpA− *E. coli* that remained bound to COS-1 cells were determined by plating on agar containing tetracycline (OmpA+ *E. coli* is sensitive to tetracycline). As shown in Figure 5A, IgG2a opsonized OmpA− *E. coli* bound COS-1 cells in significantly greater numbers compared to unopsonized bacteria and progressively more bacteria were released from the cells as more OmpA+ *E. coli* were added to the wells. In contrast, OmpA− *E. coli* could not displace bound OmpA− *E. coli*. In separate experiments, peritoneal MØ were incubated with FITC-IgG2a (1 µg) for 1 h in the presence of cytochalasin D, washed and then various quantities of OmpA+ *E. coli* or OmpA− *E. coli* were added. The cells were incubated for 10 min, washed and the amount of FITC-IgG that remained bound to the MØ was determined by flow cytometry. As shown in Figure 5B, the amount of FITC-IgG2a bound to peritoneal MØ was decreased when OmpA+ *E. coli* were added, whereas addition of OmpA− *E. coli* had no effect. These results indicate that the interaction of *E. coli* K1 with FcγRIa via OmpA can displace bound IgG2a.

Binding to the γ-chain of FcγRIa is crucial for inducing the anti-microbial activity of MØ [20]. Since OmpA binding to FcγRIa prevented the killing of the bacteria, we hypothesize that *E. coli* K1 interaction with FcγRIa avoids the association of the γ-chain. Consistent with this assumption, OmpA+ *E. coli* interaction with MØ in the presence or absence of IgG2a opsonization induced far less γ-chain association with FcγRIa in comparison to OmpA− *E. coli*, as shown by immunoprecipitation studies (Figure 5C). Similarly, OmpA+ *E. coli* induced a distinct tyrosine phosphorylation pattern of macrophage cytoplasmic proteins compared to OmpA− *E. coli* opsonized with IgG2a (Figure 5D). Taken together, these studies suggest that the interaction of *E. coli* K1 with FcγRIa can displace the bound IgG2a, which is mediated by OmpA. They also indicate that OmpA-FcγRI interaction induces novel signaling patterns, which may abrogate the normal antimicrobial response of these cells.

### FcγRIa^−/−^ mice are resistant to *E. coli* K1 infection and do not develop high degree of bacteremia

**Figure 6:**
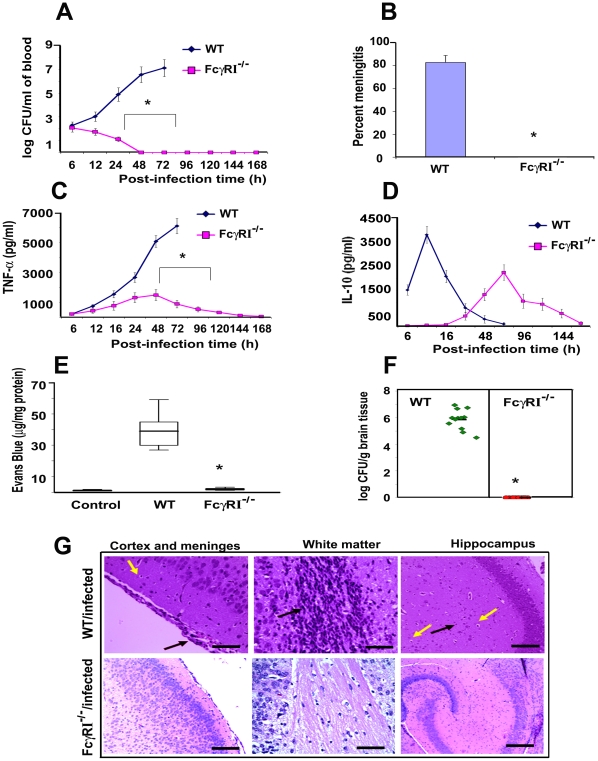
FcγRIa^−/−^ mice are resistant to *E. coli* K1 induced meningitis. (A) Wild type (WT) and FcγRIa^−/−^ mice were infected intranasally at post-natal day 3 with 10^3^ CFU of *E. coli* K1. At various time points blood was collected, dilutions made, and bacteria enumerated by plating on agar containing antibiotics. (B) Cerebrospinal fluid samples from experimental and control animals were collected and inoculated into LB broth containing antibiotics and incubated overnight at 37°C. Positive cultures indicate the occurrence of meningitis. (C and D) TNF-α and IL-10 concentrations in the blood of WT and FcγRIa^−/−^ animals infected with *E. coli* were measured by ELISA. (E) Blood-brain barrier leakage in infected animals was measured by Evans blue extravasation method as described in Materials and Methods. (F) The bacterial load in the brains of infected animals was determined by plating brain homogenates on agar containing antibiotics. (G) Brain halves from experimental and control animals were fixed, paraffin embedded, sectioned and stained with H & E. Cortex and meninges showed severe inflammation (black arrow) along with apoptosis of neurons (yellow arrow) in the brains of WT infected mice. White matter revealed increased cellularity due to inflammatory exudate (black arrow). Neutrophil infiltration (black arrow) and apoptosis of neurons (yellow arrow) were observed in hippocampus. On the contrary, no such pathological changes were seen in the brains of FcγRIa^−/−^ animals infected with *E. coli* K1. Data represent cumulative values from 15 animals in each group performed three times independently. Statistical analysis was done by Student's *t* test and Chi Square test. *p<0.001 indicates a significant difference compared with WT infected animals. Scale bars, 20 µM.

To confirm the role of FcγRIa in the pathogenesis of *E. coli* K1 meningitis, FcγRIa^−/−^ mice were used for infection studies. MØ isolated from FcγRIa^−/−^ mice did not express FcγRIa but had unchanged expression of other FcγRs, TLRs, mannose receptor and CR3 were unchanged compared to normal littermates (data not shown). The newborn animals were intranasally infected with *E. coli* K1 and examined for disease progression. Of note, the FcγRIa^−/−^ animals did not develop bacteremia even at a 100 fold higher infectious dose, even though *E. coli* K1 entered the circulation within two hours of infection (Figure S3A). In contrast, wild type (WT) animals showed 7.0 log_10_ CFU of *E. coli* K1 in blood at 72 h post-infection (Figure 6A). The FcγRIa^−/−^ mice did not develop meningitis even when infected with a 100-fold greater inoculum (data not shown). These mice did not show any signs of meningitis even after 7 days of infection, whereas 90% of WT mice showed positive CSF cultures by 72 h post-infection (Figure 6B). Cytokine analysis in the sera of these animals demonstrated that infected WT animals generated significant amounts of TNF-α, IL-1β, IL-6, IFN-γ and IL-12, but FcγRIa^−/−^ mice did not (Figure 6C and Figure S3B–D). On the other hand, IL-10 production peaked at 24 h post-infection and subsequently returned to basal levels in WT mice, whereas FcγRIa^−/−^ showed increased IL-10 production at 72 h post infection (Figure 6D). We next examined blood-brain barrier leakage in FcγRIa ^−/−^ mice. Infection with *E. coli* K1 caused no leakage in FcγRIa^−/−^ mice, whereas WT animals had significant leakage of Evans blue dye (Figure 6E). Furthermore, no bacterial colonies were detected in the brains of FcγRIa^−/−^ mice, while WT animals had a high bacterial load (Figure 6F). Similarly, the pathology of the brains from FcγRIa^−/−^ mice revealed no infiltration of neutrophils, neuronal damage or gliosis, which are the characteristic pathological features of *E. coli* K1 meningitis observed in WT bacteria infected mice (Figure 6G). In contrast, infection of FcγRIa^−/−^ mice with GBS resulted in significant bacteremia and development of meningitis (Figure S4A-C). Together these results suggest that FcγRIa expression is critical for *E. coli* K1 to achieve high-grade bacteremia and for subsequent development of meningitis in newborn mice.

### FcγRIa^−/−^ MØ exhibit greater expression of CR3 and TLR4 and produce lower levels of inducible nitric oxide

**Figure 7:**
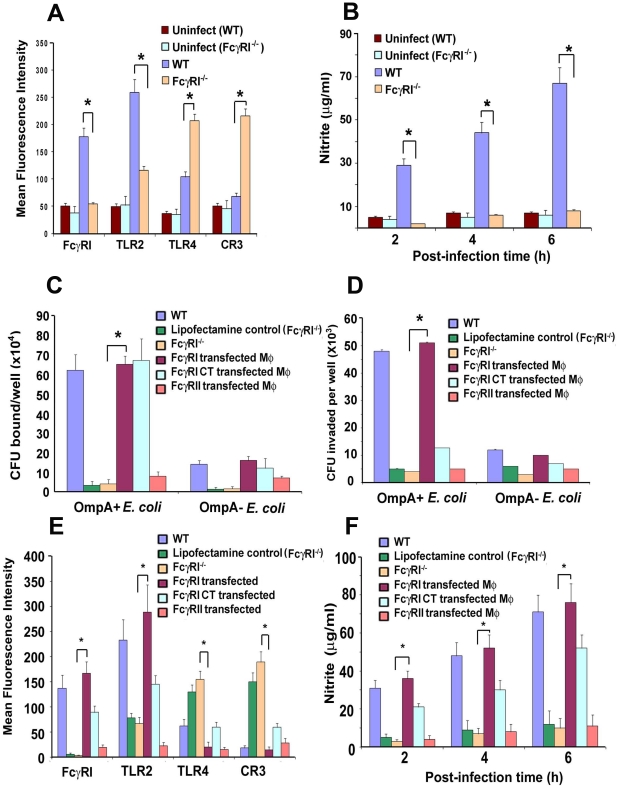
Alteration of surface receptor expression in MØ obtained from WT and FcγRIa^−/−^ mice upon infection with *E. coli* K1. (A) Peritoneal MØ from infected WT and FcγRIa^−/−^ mice were isolated, stained with antibodies to FcγRI, TLR2, TLR4 and CR3, and then subjected to flow cytometry. Data are presented after subtracting the mean fluorescence intensity (MFI) of isotype-matched control. (B) The production of NO by MØ infected with *E. coli* K1 isolated from WT and FcγRIa^−/−^ mice was measured as nitrite by the Griess method. (C and D) Bone marrow derived MØ (BMDMs) from FcγRIa^−/−^ mice were transfected with FcγRIa, FcγRIa-CT or FcγRII and used for *E. coli* K1 binding and invasion assays. (E) Flow cytometry of FcγRIa^−/−^BMDMs transfected with FcγR constructs were infected with *E. coli* K1 for 6 h, washed and then subjected to flow cytometry after staining with antibodies to FcγRI, TLR2, TLR4, or CR3. MFI values for control-uninfected cells were subtracted from the values of infected cells and then graphed. (F) NO production was also determined in FcγRI^−/−^ BMDMs transfected with FcγRIa, FcγRIa-CT or FcγRII at various times post-infection. All experiments were performed three times in triplicate. The increase or decrease in the surface expression was statistically significant by Student's *t* test, *p<0.001.

Our studies have shown that MØ isolated from *E. coli* K1 infected mice exhibit increased expression of FcγRI and TLR2, as well as increased production of nitric oxide (NO) due to iNOS activation [28]. We also observed that upregulation of CR3 expression on MØ led to enhanced killing of *E. coli* K1, whereas this effect was completely abrogated in CR3 siRNA transfected MØ *in vitro*. Other investigators have also demonstrated that CR3, TLR2 and TLR4 play important roles in the phagocytic ability of MØ [35]-[42]. Therefore, we examined whether the inability of *E. coli* K1 to survive in FcγRIa^−/−^ mice was due to altered expression of surface receptors using flow cytometry. Peritoneal MØ isolated from infected FcγRIa^−/−^ mice exhibited increased expression of CR3 and TLR4, but lower expression of TLR2 (Figure 7A). These cells also produced lower or negligible quantities of inducible NO upon challenge with *E. coli* K1, whereas MØ from WT mice generated six-fold higher amounts of NO at 6 h post-infection (Figure 7B). Furthermore, *E. coli* K1 binding to, and entry into, bone marrow-derived MØ (BMDMs) from FcγRIa^−/−^ mice were significantly lower compared to WT MØ (Figure 7C and D). Some bacteria entered FcγRIa^−/−^BMDMs, but they were killed within a short period of time as determined by immunocytochemistry (data not shown). To substantiate the role of FcγRI in *E. coli* K1 entry, FcγRIa^−/−^BMDMs were transfected with hFcγRIa, FcγRIa-CT or FcγRII and then used for binding and invasion assays. As shown in Figure 7C, *E*. *coli* K1 binding to FcγRIa^−/−^BMDM/FcγRIa and FcγRIa^−/−^BMDM/FcγRIa-CT increased significantly compared to FcγRIa^−/−^BMDMs and FcγRIa^−/−^BMDM/FcγRII. However, entry was limited to binding to FcγRIa^−/−^BMDM/FcγRIa cells only. These results suggest that FcγRIa expression is critical for *E. coli* K1 binding to, and entry into, MØ and that the C-terminal domain plays a significant role for the entry. FcγRIa^−/−^BMDM transfected with a FcγRIa construct exhibited decreased expression of TLR4 and CR3 and increased expression of FcγRI and TLR2 in comparison with FcγRIa^−/−^BMDM after challenge with *E. coli* K1 (p<0.01) (Figure 7E). Transfection with FcγRIa-CT, however, resulted in only a partial increase or decrease of these surface molecules. In contrast, FcγRIa^−/−^BMDM transfected with FcγRII showed basal level expression of these molecules. Confirming the requirement of FcγRIa interaction with *E. coli* K1 to induce NO production, FcγRIa^−/−^BMDM/FcγRIa cells generated greater quantities of NO by 6 h post-infection as compared to FcγRI^−/−^BMDM/FcγRI-CT and FcγRI^−/−^BMDM/FcγRII cells (Figure 7F). Taken together, these data suggest that FcγRIa interaction with OmpA of *E. coli* K1 is necessary for suppression of CR3 and TLR4 expression and to enhance the expression of FcγRI and TLR2, and maximal NO production.

### FcγRIa^−/−^ mice reconstituted with FcγRIa^+/+^ MØ are susceptible to *E. coli* K1 meningitis

**Figure 8:**
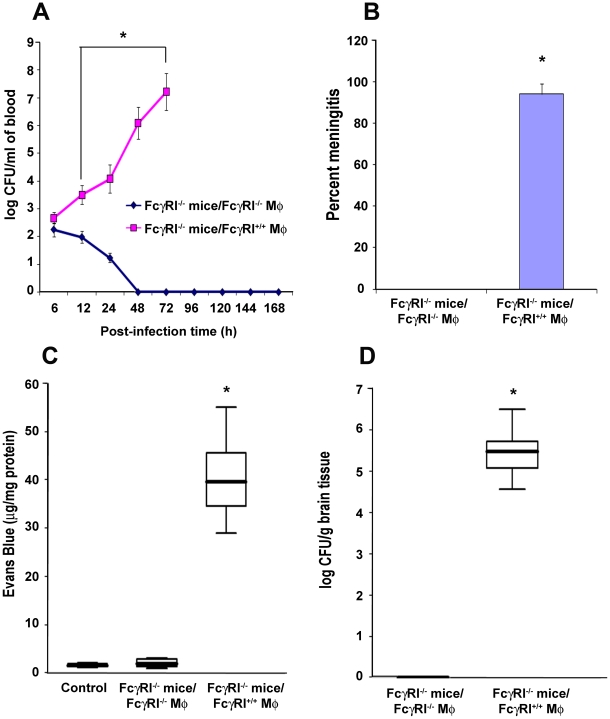
Adoptive transfer of FcγRIa^+/+^ MØ into FcγRIa^−/−^ mice restored the susceptibility to *E. coli* K1 meningitis. FcγRIa^−/−^ mice were reconstituted with FcγRIa^+/+^ MØ by intraperitoneal injection as described in Materials and Methods. Blood was withdrawn at various time points and bacteremia levels enumerated by plating the serial dilutions on agar containing antibiotics (A). Cerebrospinal fluid obtained from the same animals as described in A were directly inoculated into LB broth containing antibiotics. Positive broth cultures were considered positive for the occurrence of meningitis (B). In addition, blood brain barrier leakage (C) and brain bacterial load (D) were determined as described in Materials and Methods. Increase in these parameters in FcγRIa^+/+^ MØ reconstituted mice was statistically significant compared with FcγRIa^−/−^ MØ reconstituted animals, *p<0.001 by Student's *t* test. Results are representative of four independent experiments with 12 animals per group. Data represent mean ± SE.

To confirm the role of FcγRIa expression on MØ in the pathogenesis of *E. coli* K1 meningitis, FcγRIa^−/−^ mice were reconstituted with FcγRIa^+/+^ or FcγRIa^−/−^ MØ and then infected with *E. coli* K1. FcγRIa^−/−^ mice that received FcγRIa^+/+^ MØ showed higher blood bacterial numbers compared with animals replenished with FcγRIa^−/−^ MØ (Figure 8A). 94% of CSF cultures were positive for *E. coli* K1 in FcγRIa^+/+^ MØ reconstituted mice, whereas all cultures were sterile in animals that received FcγRIa^−/−^ MØ (Figure 8B). BBB disruption was significant in FcγRIa^+/+^ MØ-replenished animals compared to FcγRIa^−/−^ MØ reconstituted mice (Figure 8C). Higher numbers of bacteria were also recovered from the brains of mice replenished with FcγRIa^+/+^ MØ compared to animals those received FcγRIa^−/−^ MØ (Figure 8D). These results confirm that FcγRIa expression on MØ is critical for the onset of *E. coli* K1 meningitis.

## Discussion

The host response to infection starts with the identification of invading microorganisms via innate immune surveillance systems [43]. Nonetheless bacterial pathogens utilize very effective mechanisms to avoid host defenses in order to promote successful replication and dissemination [44]. MØ provide an important innate and adaptive immune coverage in the host, although their importance in *E. coli* K1 meningitis is unexplored. In the present study, we demonstrate that the expression of FcγRIα-chain in MØ is critical for the survival of *E. coli* K1 inside these immune cells by using MØ-depleted and FcγRIa^−/−^ mice. It is tempting to speculate that the ability of *E. coli* K1 to survive inside MØ might enable these bacteria to infect the central nervous system via a “Trojan horse” mechanism. Pathogens that naturally infect the central nervous system, such as *Brucella*, *Listeria*, and *Mycobacterium*, have been demonstrated to use this mode of entry [45], [46]. We observed that the interaction of OmpA with FcγRIa in MØ is critical for bacterial binding to, entry into, and subsequent survival in these cells. Generally various FcγRs recognize microbes coated with either specific or non-specific antibodies. However a select number of microbes have developed methods to avoid this recognition. Protein A of *S. aureus* is known to bind to the Fc portion of the antibodies so that it avoids interacting with FcγRI, whereas most other microbes either downregulate phagocytic mechanisms or avoid phagocytosis entirely [47], [48]. This study therefore depicts the first evidence that a bacterial protein binds directly to FcγRIa to divert anti-microbicidal mechanisms.

Our competitive inhibition studies demonstrated that OmpA interacts with FcγRIa and can displace the binding of Fc portion of IgG. Therefore, it is possible that the bacteria in circulation, despite being coated with non-specific IgG, interact with MØ via FcγRIa for binding to and entering the cells for subsequent multiplication. OmpA− *E. coli* could not survive in MØ, suggesting that the interaction of OmpA with FcγRIa induces survival strategies or suppresses anti-microbial pathways in MØ. However, OmpA− *E. coli* has been shown to express reduced levels of type 1 fimbriae and susceptible to chemical stresses [49], [50]. Therefore, it is possible that OmpA− *E. coli* could be less capable of dealing with macrophage-induced stresses. *Listeria, Shigella*, and *Rickettsia* escape from the phagosome to the cytosol to avoid destruction in phagolysosomes [51]. Other pathogens interfere with the normal biogenesis of phagolysosomes, thus leading to the formation of replicative vacuoles [52], [53]. Since *E. coli* K1 continue to multiply inside phagosomes, one can speculate that phagosomes containing OmpA+ *E. coli* avoid lysosomal fusion by blocking phagosome maturation. The receptors expressed on the surface of MØ play a decisive role in the course of infection, whether pathogens are killed or the MØ machinery is taken over by the microbes [54]. Receptors like TLR2, TLR4 and CR3 have been implicated in the phagocytic ability of MØ [55], [56], [57]. Downregulation of CR3 expression on the surface of MØ has been associated with the decrease in the phagocytosis of pathogens and hence survival inside MØ [58]. TLR2 expression has been shown to prolong survival of *Staphylococcus aureus* inside phagosomes in MØ, which may be a strategy adopted by this pathogen to evade innate immunity. On par with this concept, TLR2 or MyD88 KO mice have been demonstrated to be resistant to sepsis, indicating that TLR2 mediated signaling is playing an important role in the survival of bacterial pathogens [59]. Activation of MØ through TLR4 has been shown to direct the induction of Th1 and Th-17 cells, which mediate protective cellular immunity to *Bordetella pertussis* by enhancing the bactericidal activity of MØ [60]. It is still to be determined whether TLR2 expression upon *E. coli* K1 infection has any role in the pathogenesis of meningitis.

We recently demonstrated that iNOS^−/−^ mice and aminoguanidine (iNOS specific inhibitor) treated MØ showed enhanced expression of CR3 and TLR4 and very low levels of TLR2 and FcγRI, indicating that iNOS suppression results in decreased expression of FcγRI [28]. In agreement with these studies, we showed here that lack of FcγRI in MØ prevented the production of inducible NO and increased the expression of CR3 and TLR4, indicating that OmpA-FcγRIa interaction is critical for manipulating the surface expression of CR3 and TLRs in MØ. Our current results indicate that in *E. coli* K1 pathogenesis, FcγRI interaction with OmpA enhances the expression of TLR2, which in turn can be utilized by the bacteria as a receptor to modulate the efficiency of phagosome formation. Alternatively, *E. coli* K1 interaction with FcγRIa activates non-microbicidal mechanisms for the bacterial survival in MØ. Our studies have demonstrated that *E. coli* K1 infected MØ also exhibit increased expression of gp96, a known chaperone for TLR2 and TLR4 [28]. These interactions may also induce effector proteins into MØ by *E. coli* K1 that eventually are responsible for the control of macrophage environment. Further studies are in progress to examine these possibilities. As cytokines are known to modulate MØ microbicidal activity, it is also possible that the surface expression of TLRs and CR3 could be controlled by the circulating cytokines in *E. coli* K1 infection. Of note, we have demonstrated that IL-10 administration suppressed the expression of FcγRI and enhanced the expression of TLR4 and CR3, which in turn prevented the survival of *E. coli* K1 in MØ [61]. In contrast, for several other pathogens, circulating IL-10 supports intracellular replication, indicating that *E. coli* K1 pathogenesis is distinct from that induced by other bacterial pathogens [62].

Previous studies have shown that the cytoplasmic (CY) domain of FcγRIa plays an important role in phagocytosis and antigen presentation [63]. However, lack of the CY domain neither alters the association of γ-chain with FcγRIa nor influences the tyrosine phosphorylation of γ-chain in response to receptor specific cross-linking [63]. In contrast to these findings, we observed that OmpA binding to FcγRIa did not induce the association of γ-chain despite the presence of the CY domain. This binding also induced a different tyrosine phosphorylation response in MØ. Therefore, the CY domain of FcγRIa induces signaling events independent of γ-chain during the invasion of *E. coli* K1. Similarly, Qin et al demonstrated that the CY domain induces different gene expression in murine MØ compared to MØ stably transfected with CY-deleted FcγRIa [64]. Alteration of signal transduction pathways to impair FcγR-mediated phagocytosis has also been observed in HIV infected MØ, which have downregulated the expression of the γ-subunit [65]. Moreover, direct interaction of periplakin with the CY domain of human FcγRIa can confer unique properties on this receptor [66]. It should be noted that there are significant differences in the cytoplasmic regions of human and murine FcγRIa. However, our data demonstrate that the interaction of OmpA induced a similar response in both human and murine MØ. In summary, our studies provide the first evidence that a bacterial protein interacts directly with FcγRIa in order to bind to and enter MØ and manipulates the intracellular signaling for bacterial survival and multiplication. The new repertoire of interaction also suggests that MØ function may be manipulated by targeting additional epitopes without activating MØ microbicidal function. This strategy will be useful for devising novel methods of therapy for other diseases involving FcγRIa in addition to neonatal *E. coli* K1 meningitis.

## Materials and Methods

### Ethics statement

This study was carried out in strict accordance with the recommendations in the Guide for the Care and Use of Laboratory Animals of the National Institutes of Health. The protocol was approved by the Institutional Animal care and Use Committee (IACUC) of The Saban Research Institute of Childrens Hospital Los Angeles (Permit number: A3276-01). All surgery was performed under sodium pentobarbital anesthesia, and all efforts were made to minimize suffering.

### Bacteria

*E. coli* E44, a rifampin-resistant mutant of *E. coli* K1 strain RS 218 (serotype O18:K1:H7), has been isolated from the cerebrospinal fluid of a neonate with meningitis and invades human brain microvascular endothelial cells (HBMEC) [34]. E91, a derivative of E44 in which *ompA* gene is disrupted (designated as OmpA− *E. coli*) and HB101 (a laboratory *E. coli* strain that expresses K-12 capsular polysaccharide) are noninvasive in HBMEC [34]. Group B *streptococcus* type III strain COH-1 used in these studies was provided by Dr. Craig Rubens of Seattle Children's Hospital, Seattle [67]. All bacteria were grown in brain heart infusion broth with appropriate antibiotics as necessary. Bacterial media were purchased from Difco laboratories (Detroit, MI).

### Cell culture and reagents

Murine MØ cell line RAW 264.7, human macrophage like cells, THP-1 and COS-1 cells were obtained from American Type Culture Collection (Manassas, VA). COS-1 cells were stably transfected with cDNA encoding human FcγRIa, a mutant form of FcγRIa containing a stop codon after first amino acid of the cytoplasmic domain (Lys^315^→Stop 315) (FcγRI-CT), or with human FcγRII [67]. Anti- FcγRI (blocks the binding of Fc-portion of IgG to FcγRI), anti-CD11b, anti-CD32, anti-MR, anti-TLR2, anti-TLR4 and anti-Myc antibodies were obtained from Cell signaling. Purified IgG2a and FITC-IgG2a were obtained from Sigma (St. Louis, MO). Anti-gp96 antibody was raised in our lab as previously described [34], [68]. Anti-phospho-tyrosine antibody (4G10) was obtained from BD Sciences and all secondary antibodies coupled to various fluorophores were obtained from Bio-Rad Labs (Hercules, CA).

### Bacterial invasion assays

Confluent MØ monolayers in 24-well plates were incubated with 1×10^6^ *E. coli* K1 (multiplicity of infection of 10) in experimental medium (1:1 mixture of Ham's F-12 and M-199 containing 5% heat-inactivated fetal bovine serum) for 60°min at 37°C, whereas COS-1 cell monolayers were infected with *E. coli* K1 at an MOI of 100 for 1.5 h. The monolayers were washed three times with RPMI 1640°and further incubated in experimental medium containing gentamicin (100 µg/ml) for 1 h to kill extracellular bacteria. The monolayers were washed again and lysed with 0.5% Triton X-100. The intracellular bacteria were enumerated by plating on sheep blood agar. In duplicate experiments, the total cell associated bacteria were determined as described for invasion except that the gentamicin step was omitted.

### Generation of FcγRIa^−^ and CR3^−^ RAW 264.7 cells

SureSilencing shRNA plasmids to mouse FcγRIa and CR3 (CD11b) in the pGeneClip Neomycin Vector were obtained from Super Array Inc., (Frederick, MD). RAW 264.7 cells were transfected with shRNA plasmids using Lipofectamine 2000 and later selected for G418 resistant colonies.

### Biotinylation of MØ membrane proteins

The cell surface proteins of THP-1 cells differentiated into MØ (THP-M) and RAW 264.7 cells were biotinylated by adding to 0.1 M sodium bicarbonate buffer (pH 8.0) containing 0.5 mg/ml NHS-LC-Biotin (Pierce Co, Rockford, IL) at a final protein concentration of 2 mg/ml in tissue culture flasks. The flasks were incubated on ice for 1 h, the cells were extensively washed with ice-cold PBS and solubilized in 5% Triton X-100 in PBS. Total membranes from the cells were isolated following extensive dialysis against PBS and then were concentrated using Centricon tubes (Millipore, Bedford, MA; 10-kDa cut-off). Biotinylated proteins (2–5 µg) were incubated with various bacteria from a 5-ml overnight culture in a volume of 0.5 ml at 37°C on a rotator for 1 h. The bacteria were then centrifuged and the pellets were washed three times with PBS containing 0.1% Triton X-100. After a final wash, the bound proteins were released with Laemmli buffer in the presence of β-mercapto-ethanol and analyzed by SDS-PAGE. The separated proteins were transferred to nitrocellulose and immunoblotted with streptavidin coupled to peroxidase. The protein bands were visualized by ECL reagent (Amersham Biosciences, Piscataway, NJ).

### RNA isolation and RT-PCR

Total RNA was isolated from various transfected RAW 267.4 cells with TRIZOL-LS-reagent (Gibco BRL, Gaithersburg, MD) and quantified using a nanodrop machine. RT-PCR was performed using the following primer sequences: FcγRIa (321 bp) FP 5′-TCCTTCTGGAAAATACTGACC-3′ and RP 5′ GTTTGCTGTGGTTTGAGACC-3′; TLR2 (459 bp) FP 5′-TGAGAGTGGGAAATA TGGAC-3′; RP 5′-CCTGGCTCTATAACTCTGTC-3′; TLR4 (506 bp) FP 5′- TGGAT ACGTTTCCTTATAAG-3′ and RP 5′-GAAATGGAGGCACCCCTTC- 3′; GAPDH (479 bp), FP 5′-CACAGTCCATGCCATCACTG-3′ and RP 5′- TACTCCTTGGAG GCCATGTG -3′. Negative control assays without primers were performed in parallel for every reaction. The amplified products were separated on a 1% agarose gel and were stained with ethidium bromide.

### Flow cytometry

Expression of FcγRI, CR3, TLR2 and TLR4 was detected by staining with appropriate FITC-, phycoerythrin (PE)-, PE-CY5.5-, or allophycocyanin (APC)-coupled mouse monoclonal antibodies (eBiosciences, San Diego, CA). Cells were first pre-incubated for 20 min with IgG blocking buffer to mask non-specific binding sites and then further incubated with the indicated antibodies or an isotype control antibody for 30 min at 4°C. The cells were subsequently washed three times with PBS containing 2% FBS and fixed with BD Cytofix (BD Biosciences). Cells were then analyzed by four-color flow cytometry using FACS calibur Cell Quest Pro software (BD Biosciences, San Jose, CA). Side and forward scatter parameters for which F4/480 was used as a MØ-gating marker, which formed the collection gate and at least 5000 events within this gate were collected for analysis.

### Depletion of MØ

Newborn C57BL/6 mice were injected intraperitoneally with (20-mg/Kg body weight) α-carrageenan (Sigma, St. Louis, MO) on days 1, 2 and 3 before infecting with *E. coli*. In control groups, mice were treated with equal volumes of saline.

### Newborn mouse model of meningitis

Three-day old mice were randomly divided into various groups and infected intranasally with 10^3^ CFU of bacteria. Control mice received pyrogen free saline through the same route. Blood was collected from the tail or facial vein at designated times post-infection and plated on LB agar containing rifampicin to assess bacteremia and level of infection. CSF samples were collected aseptically under anesthesia by cisternal puncture and directly inoculated into broth containing antibiotics. Mice were perfused intracardially with 0.9 % saline to remove blood and contaminating intravascular leukocytes. Brains were aseptically removed and homogenized in sterile PBS. Bacterial counts in all tissues were determined by plating ten-fold serial dilutions on rifampicin LB agar plates. Growth of *E. coli* in rifampicin containing LB broth from the CSF samples was considered positive for meningitis [28].

### Characterization of liver and spleen leukocytes

Determination of leukocytes in livers and spleens of untreated and carrageenan treated mice was done using flow cytometry [61]. PMNs were identified by staining with anti-Ly6-G (GR-1) followed by goat anti-rat- phycoerythrin (PE). CD4^+^ and CD8^+^ T lymphocytes were stained with rat anti-mouse-CD4 followed by goat anti-rat-PE and anti-CD8-FITC. DCs were stained with APC conjugated anti-CD11c antibody. B lymphocytes were detected by staining with anti-CD45R (B220)-FITC. Flow cytometry was performed on a FACScan instrument (BD Biosciences, CA) and the data were analyzed with Cell Quest Software.

### Immunoprecipitation and Western blotting

Total cell lysates of RAW 264.7 cells infected with bacteria for varying time periods were centrifuged at 16,000 X *g* for 20 min at 4°C. The supernatants were collected and the protein contents determined. For immunoprecipitation studies, 300–500 µg of protein was incubated with the appropriate antibody overnight at 4°C, washed and further incubated for 1 h with protein A-agarose. The immune complexes were washed four times with cell lysate buffer and the proteins bound to agarose were eluted in SDS sample buffer for further analysis by Western blotting. Portions of the cell lysates were subjected to electrophoresis on a 10% SDS-polyacrylamide electrophoresis gel. The proteins were transferred to a nitrocellulose membrane, which was then blocked with 5% bovine serum albumin (BSA) in Tris-buffered saline containing 0.05% Tween 20 (TBST) for 2 h at room temperature. The blot was then incubated with the primary antibody overnight at 4°C in 5% BSA/TBST. The blot was washed with TBST and further incubated with the horseradish peroxidase-conjugated secondary antibody for 1 h at room temperature. Subsequently, the blot was washed four times with TBST for 1 h, developed with SuperSignal chemiluminescence reagent, and exposed to x-ray film to visualize the proteins.

### Transmission electron microscopy

RAW 264.7 cells were incubated with *E. coli* K1 at an MOI of 10 for varying times, washed and then fixed with 2% glutaraldehyde in 0.1 M cacodylate buffer, pH 7.1. All samples were washed three times in 0.1 M cacodylate buffer for 15 minutes each. The cells were then post-fixed for 20 minutes in 1% osmium tetroxide at 4°C followed by addition of EtOH (60%). Samples were dehydrated through 70, 80, 95, and 100% EtOH (two times, 15 min each), then into propylene oxide (two time, 15 min each), and into a 1:1 propylene oxide/Eponate, left overnight, capped, at room temperature. The propylene oxide/Eponate mixture was decanted off and replaced with 100% Eponate mixture. The samples were polymerized at 70°C for 48 h. Thin sections (∼80 nm) were cut using a diamond knife, mounted on un-coated 300 mesh copper grids and stained with 5% uranyl acetate for 20 min. Photographs were take with a transmission electron microscope (JEOL JEM 2100 LaB6) equipped with a Gatan Ultra Scan 1000 CCD camera.

### Competitive binding assays

COS-1 cells were grown in 24-well tissue culture plate to confluence and then treated with 0.5 µg/ml of cytochalasin D for 30 min prior to addition of bacteria. OmpA− *E. coli* were incubated with anti-S-fimbria antibody for 1 h on ice, washed, and then added to the COS-1 monolayers at an MOI of 100 for 1 h. OmpA− *E. coli* alone infected monolayers served as controls in these experiments. The monolayers were then washed to remove unbound bacteria and incubated with OmpA+ *E. coli* at an MOI of 10 and 100 for 10 min, washed the monolayers, and then dissolved in 150 µl of PBS containing 0.3% Triton X-100. Serial dilutions were made and plated on agar containing tetracycline (12.5 µg/ml) in which only OmpA− *E. coli* grow. The number of CFU was counted and determined the percent displacement by OmpA+ *E. coli*. In some experiments, FITC-IgG2a (1 µg) was incubated with cytochalasin-D treated peritoneal MØ while rotating the test tube at a low speed for 30 min and washed to remove unbound IgG. Various inocula of OmpA+ *E. coli* or OmpA− *E. coli* were added to the cells and incubated for 10 min, washed and the bound FITC-IgG was determined by flow cytometry.

### Differential staining of *E. coli* K1

RAW 264.7 cells were grown in eight-well chamber slides and infected with *E. coli* K1 as described above. The monolayers were then washed with PBS and fixed in 2% paraformaldehyde for 10 min at room temperature. Subsequently, anti-S-fimbria antibody (1:1000 dilution) was added to the cells and incubated for 1 h at room temperature. The cells were then washed with PBS and incubated with secondary antibodies conjugated to FITC for 30 min at room temperature. The monolayers were washed four times with PBS and incubated with excess amounts of secondary antibody coupled to horseradish peroxidase for 1 h at RT to block the external primary antibody sites. After thorough washing of the cells, the monolayers were permeabilized with 5% normal goat serum in phosphate-buffered saline containing 1% Triton X-100 (NGS/PBST) for 30 min. The cells were again incubated with anti-S-fimbria antibody for 1 h in Triton/NGS/PBST buffer, washed and further incubated with secondary antibody coupled to Cy3 for 30 min. The cells were washed again, the chambers removed, and the slides mounted in Vectashield (Vector Laboratories) anti-fade solution containing 4′, 6-diamidino-2-phenylindole. Cells were viewed using a Leica (Wetzlar, Germany) DMRA microscope with Plan-apochromat ×40/1.25 NA and ×63/1.40 NA oil immersion objective lenses. Image acquisition was with a SkyVision-2/VDS digital CCD (12-bit, 1280×1024 pixel) camera in unbinned or 2×2-binned models into EasyFISH software, saved as 16-bit monochrome, and merged as 24-bit RGB TIFF images (Applied Spectral Imaging Inc., Carlsbad, CA). The images were assembled and labeled using Adobe PhotoShop 7.0.

### Determination of the BBB leakage

BBB permeability was quantitatively evaluated by detection of extravasated Evans blue dye [28]. Briefly, 2% Evans blue dye in saline was injected intraperitoneally into infected or uninfected mice and after 4 h, mice were deeply anesthetized with Nembutal and transcardially perfused with PBS until colorless perfusion fluid was obtained from the right atrium. Brains from infected animals were harvested, weighed and homogenized. Tissue supernatant was obtained by centrifugation and protein concentration was determined. Evans blue intensity was determined on a microplate reader at 550 nm. Calculations were based on external standards dissolved in the same solvent. The amount of extravasated Evans blue dye was quantified as micrograms per milligram protein.

### Isolation of peritoneal MØ and adoptive transfer of MØ

Peritoneal MØ were isolated from mice according to the method of Mittal et al [28], [69], [70]. Briefly, the mouse peritoneal cavity was exposed carefully without disrupting blood vessels and 2–3 ml of RPMI was slowly injected. The lavage was collected and cultured in tissue culture flasks for 2 h at 37°C under 5% CO_2_ to allow adherence of MØ. Non-adherent cells were removed and the flasks washed three times with Hanks' solution. The adherent cells were harvested from the flasks using a rubber policeman and were resuspended in 10% FCS-RPMI 1640 medium. MØ were then positively selected using Miltenyi biotech kit and percentage purity examined by FACS analysis using F4/80 antibody, which was >97%. Viability of MØ following interaction with bacteria was assessed using an Annexin V kit (BD Biosciences, San Diego, CA). Mouse bone marrow cells were isolated from the tibias and femurs of 6- to 10-wk-old WT and FcγRI^−/−^ mice [71]. After euthanasia of mice by CO_2_ asphyxiation, femurs were harvested and bone marrow cells aseptically flushed from the marrow cavities with ice-cold PBS. Cells were collected by centrifugation and erythrocytes were lysed by resuspending in 0.15 M NH_4_Cl for 3–5 min. Celle were washed with PBS and resuspended in complete DMEM medium supplemented with M-CSF (10 ng ml^−1^) and IL-3 (10 ng ml^−1^), plated and allowed to differentiate into MØ. After 5–7 days in culture, adherent MØ were washed with PBS, scraped gently into suspension and counted. The purity of the MØ was determined by flow cytometry using F4/80 antibody and found to be >95%. Fresh bone marrow derived MØ (5×10^6^ cells) were transferred by intraperitoneal injection into mice 6 h before infecting with *E. coli* K1.

### Estimation of NO production

NO production was determined in MØ supernatants by a modified Griess method as described earlier [28], [72], [73]. Briefly nitrate was converted to nitrites with β-nicotinamide adenine dinucleotide phosphate (NADPH, 1.25 mg ml^−1^) and nitrate reductase followed by addition of Griess reagent. The reaction mixture was incubated at room temperature for 20 min followed by addition of TCA. Samples were centrifuged, clear supernatants were collected and optical density was recorded at 550 nm. The amounts of NO produced were determined by calibrating standard curve using sodium nitrite.

### Histopathology

Half of the brain was fixed in 10% buffered formalin, routinely processed and embedded in paraffin. 4–5 µm sections were cut using a Leica microtome and stained with hematoxylin and eosin (H & E). Pictures were taken using a Zeiss Axiovert Microscope connected to a JVC 3-chip color video camera and read by the pathologist in a blinded fashion.

### Cytokine assays

Cytokine (TNF-α, IL-1β, IL-6, IL-12 p70 and IL-10) levels in sera from various animals were determined using Biosource ELISA kits (Invitrogen, Carlsbad, CA) according to the manufacturer's instructions.

### Statistical analysis

For statistical analysis of the data, two tailed Fischer test, Wilcoxon signed rank test and Student's *t*-test were applied and p value <0.05 was considered statistically significant.

## Supporting Information

Figure S1 Analysis of various cell types in MØ-depleted mice. MØ were depleted in newborn mice by the administration of carrageenan as described in Materials and Methods. Spleens and livers were harvested, homogenized, and the cells in the homogenates were subjected to flow cytometry for analysis of neutrophils (A), dendritic cells (DCs) (B), CD4+ T cells (C), CD8+ T cells (D) and B cells (E).
(3.58 MB TIF)

Figure S2 Cytokine production in MØ-depleted mice infected with *E. coli* K1. WT and MØ-depleted newborn mice were infected with 10^3^ CFU of *E. coli* K1 by intranasal instillation, blood samples were collected at various times, and the concentrations of IL-β (A), IL-6 (B), IFN-γ (C) and IL-12 (D) determined by ELISA as described in Materials and Methods. The data represent means ± SD of three independent experiments with five animals in each group. The decrease in the cytokines in MØ-depleted animals was statistically significant compared to WT animals, *p<0.001 by Student's *t* test.
(2.54 MB TIF)

Figure S3 Bacteremia and cytokines levels in WT and FcγRIa^−/−^mice infected with *E. coli* K1. (A) WT and FcγRI^−/−^ mice at day 3 were infected with *E. coli* K1, blood samples collected at various times, dilutions made and plated on blood agar containing antibiotics. The levels of IL-1β (B), IL-6 (C), IL-12 (D) in the blood samples were determined by ELISA. The data represent means ± SD of three separate experiments performed in triplicate with fifteen animals in each group.
(1.28 MB TIF)

Figure S4 Bacterial load and the occurrence of meningitis in newborn mice infected with Group B streptococcus. (A) WT and FcγRIa^−/−^ mice at day 3 after birth were infected with 10^5^ CFU of GBS intranasally. Blood was collected at 24, 48 and 72 h post-infection, dilutions were made, and plated on agar. (B) CSF samples were collected aseptically by cisternal puncture and inoculated directly into LB broth, and positive CSF cultures were considered positive for the occurrence of meningitis. (C) At 72 h post-infection brains were harvested and half of the brains were homogenized in PBS, dilutions were made and plated on agar. The data represent mean ± SD of three separate experiments with four animals each group.
(0.71 MB TIF)
