# Supplementary figures and images for: Fcγ Receptor I Alpha Chain (CD64) Expression in Macrophages Is Critical for the Onset of Meningitis by Escherichia coli K1
Source: PLoS Pathog. 2010 Nov 18;6(11):e1001203. doi: 10.1371/journal.ppat.1001203 (PMC2987830; doi:10.1371/journal.ppat.1001203)

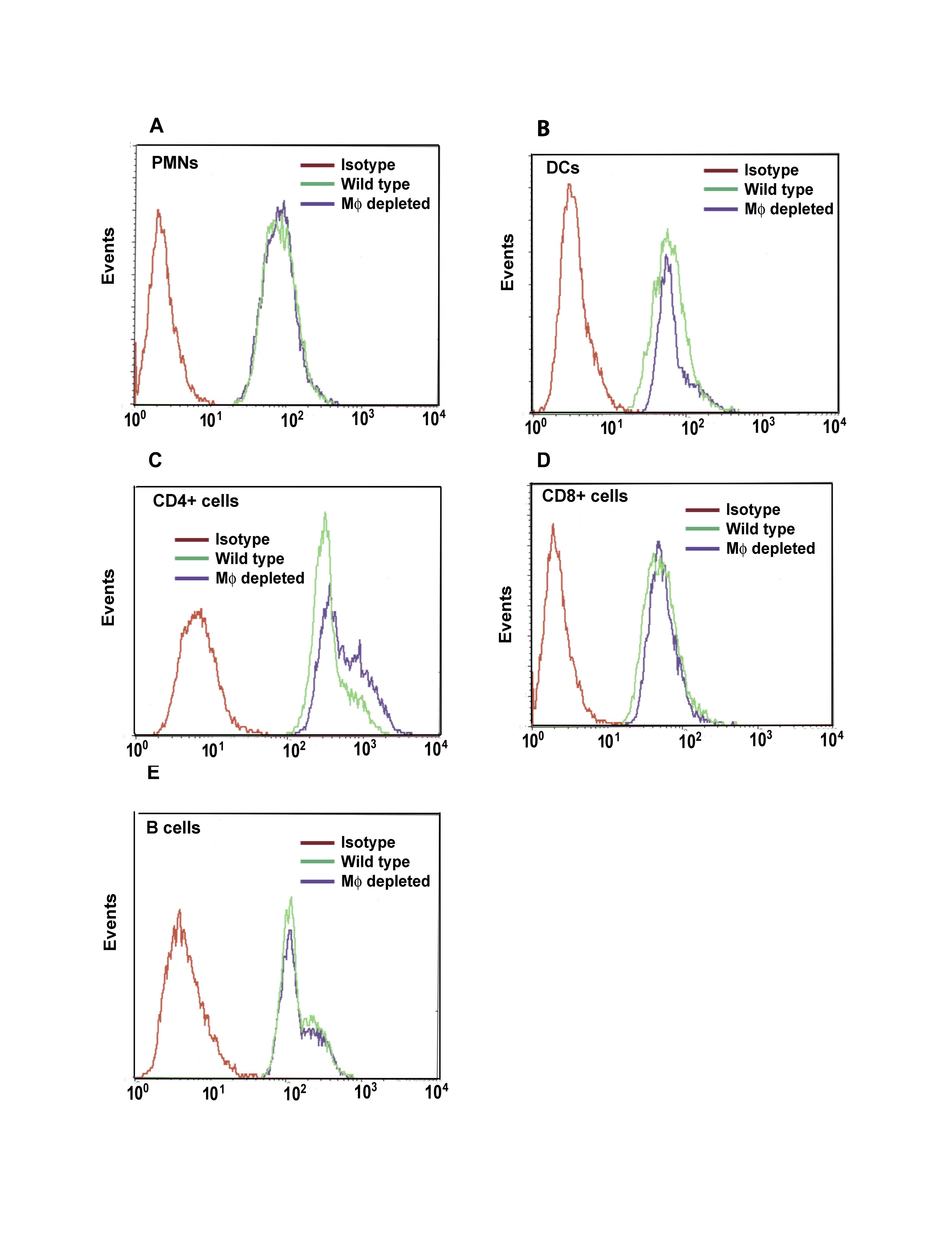

Supplement: Figure S1 — Analysis of various cell types in MØ-depleted mice. MØ were depleted in newborn mice by the administration of carrageenan as described in Materials and Methods. Spleens and livers were harvested, homogenized, and the cells in the homogenates were subjected to flow cytometry for analysis of neutrophils (A), dendritic cells (DCs) (B), CD4+ T cells (C), CD8+ T cells (D) and B cells (E). (3.58 MB TIF) [file ppat.1001203.s001.tif]

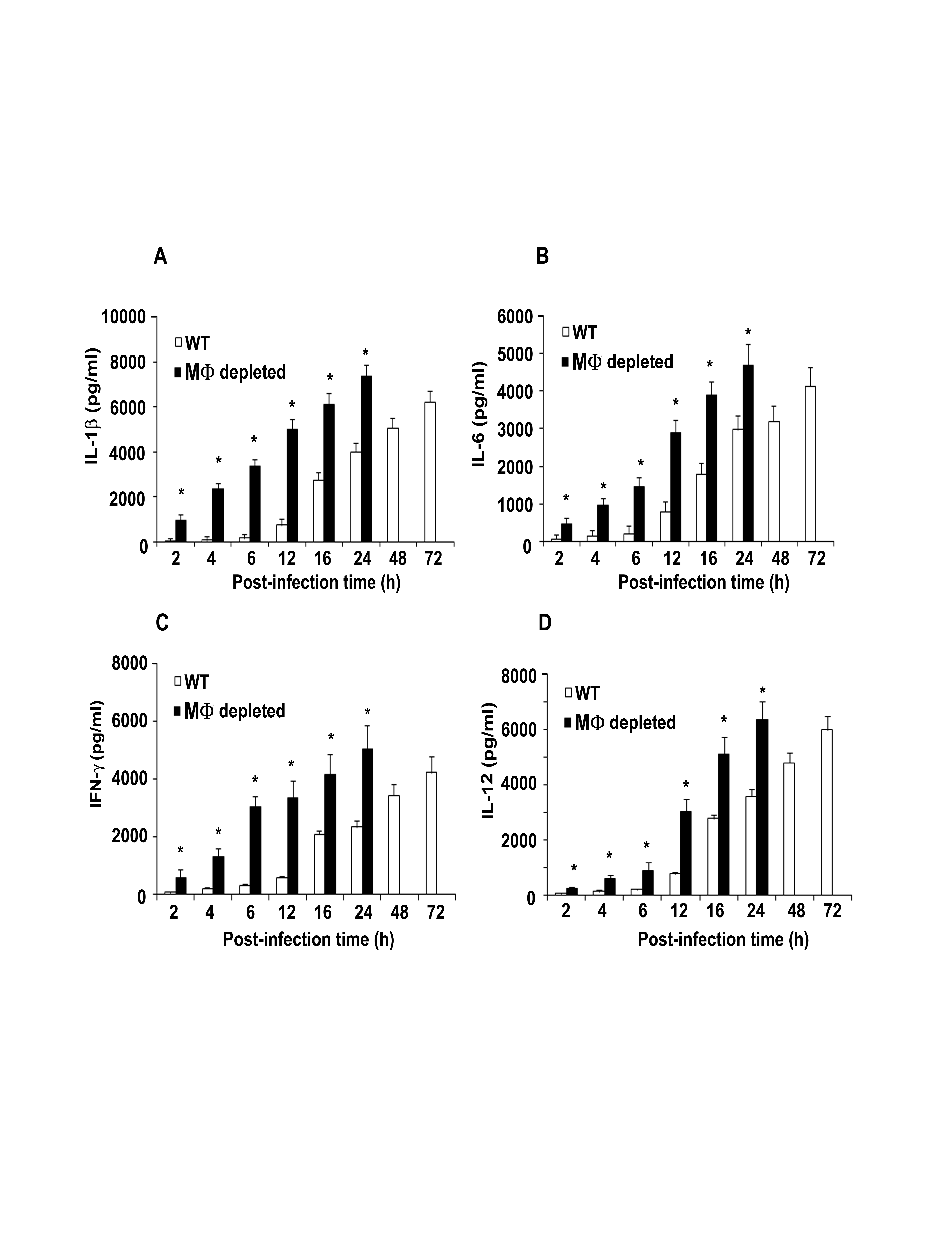

Supplement: Figure S2 — Cytokine production in MØ-depleted mice infected with E. coli K1. WT and MØ-depleted newborn mice were infected with 103 CFU of E. coli K1 by intranasal instillation, blood samples were collected at various times, and the concentrations of IL-β (A), IL-6 (B), IFN-γ (C) and IL-12 (D) determined by ELISA as described in Materials and Methods. The data represent means ± SD of three independent experiments with five animals in each group. The decrease in the cytokines in MØ-depleted animals was statistically significant compared to WT animals, *p<0.001 by Student's t test. (2.54 MB TIF) [file ppat.1001203.s002.tif]

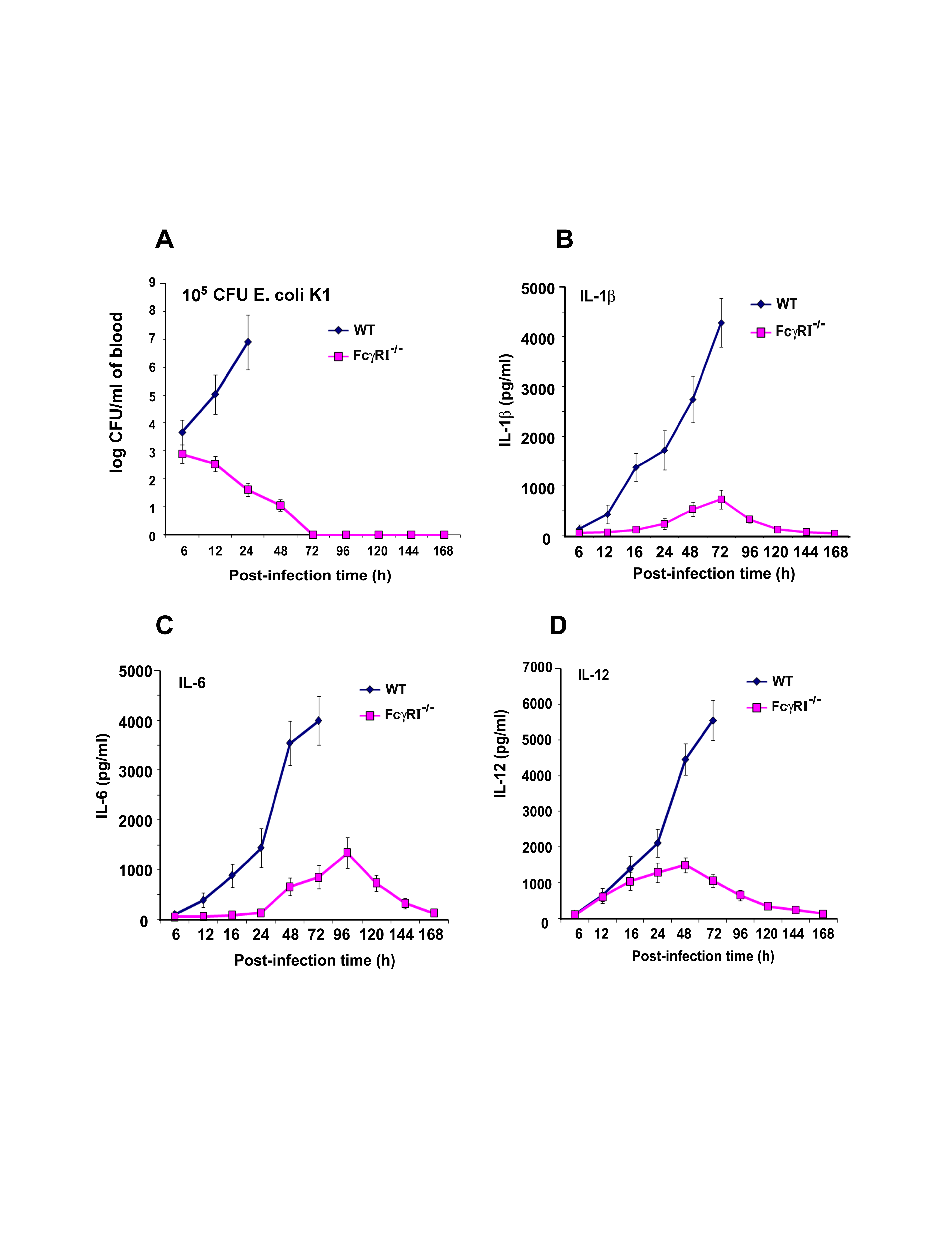

Supplement: Figure S3 — Bacteremia and cytokines levels in WT and FcγRIa−/−mice infected with E. coli K1. (A) WT and FcγRI−/− mice at day 3 were infected with E. coli K1, blood samples collected at various times, dilutions made and plated on blood agar containing antibiotics. The levels of IL-1β (B), IL-6 (C), IL-12 (D) in the blood samples were determined by ELISA. The data represent means ± SD of three separate experiments performed in triplicate with fifteen animals in each group. (1.28 MB TIF) [file ppat.1001203.s003.tif]

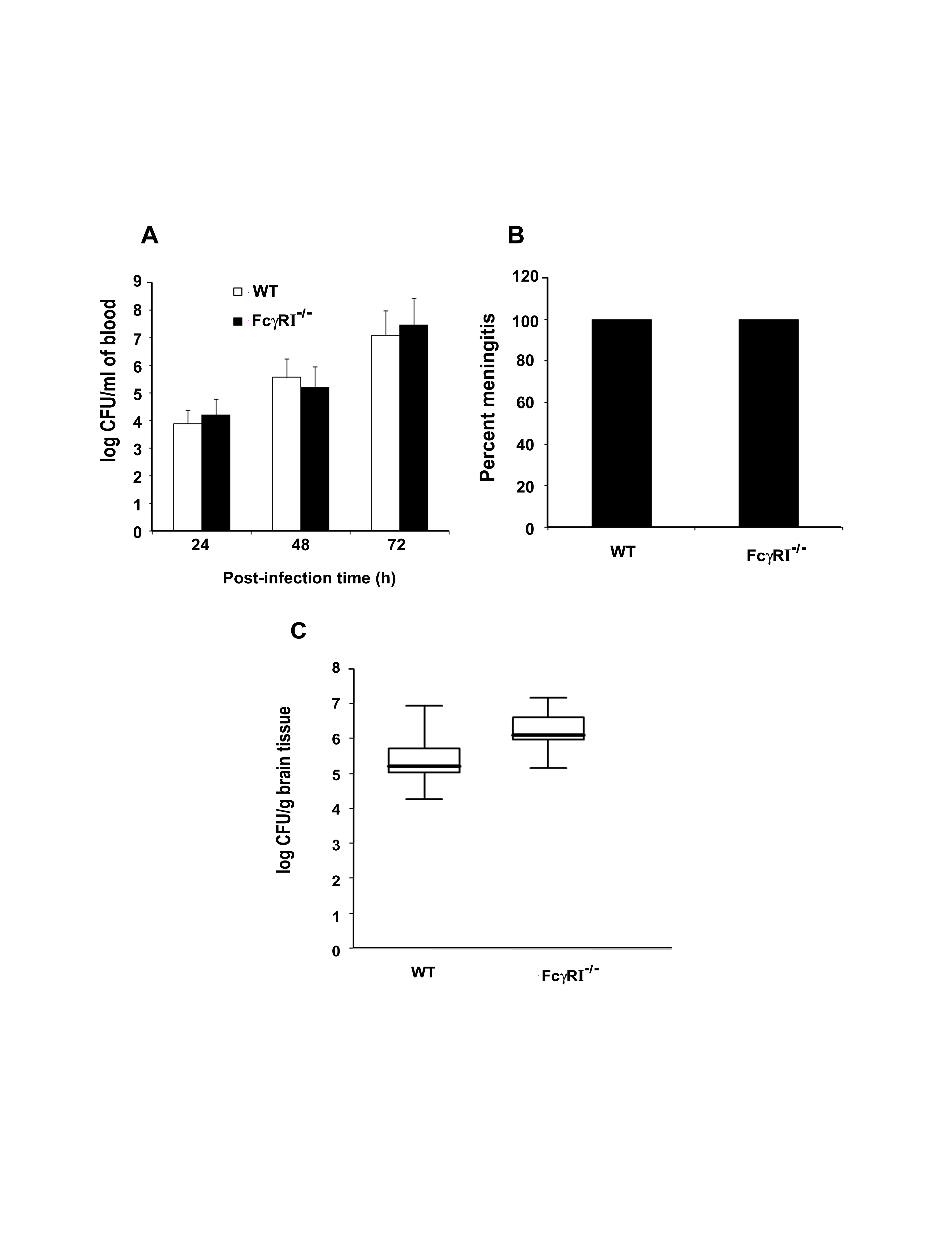

Supplement: Figure S4 — Bacterial load and the occurrence of meningitis in newborn mice infected with Group B streptococcus. (A) WT and FcγRIa−/− mice at day 3 after birth were infected with 105 CFU of GBS intranasally. Blood was collected at 24, 48 and 72 h post-infection, dilutions were made, and plated on agar. (B) CSF samples were collected aseptically by cisternal puncture and inoculated directly into LB broth, and positive CSF cultures were considered positive for the occurrence of meningitis. (C) At 72 h post-infection brains were harvested and half of the brains were homogenized in PBS, dilutions were made and plated on agar. The data represent mean ± SD of three separate experiments with four animals each group. (0.71 MB TIF) [file ppat.1001203.s004.tif]
